# Anti-CD31 antibody preconditioning for enhancement of endothelial cell capture and vascularization: a novel strategy for bioengineering lung scaffolds

**DOI:** 10.1186/s13036-025-00593-x

**Published:** 2026-01-08

**Authors:** Satoshi Kamata, Arash Zargar, Daisuke Taniguchi, Yamato Suzuki, Abbie Lo, Shiyuan Bian, Ethan Chen, Samantha Ligi, Shaf Keshavjee, Yoshinori Okada, Siba Haykal, Aimy Bazylak, Thomas K. Waddell, Golnaz Karoubi

**Affiliations:** 1 Latner Thoracic Research Laboratories, Division of Thoracic Surgery, Toronto General Hospital Research Institute, University Health Network, University of Toronto, Toronto, Canada; 2 Department of Thoracic Surgery, Institute of Development, Aging and Cancer, Tohoku University, Sendai, Japan; 3 Department of Mechanical and Industrial Engineering, University of Toronto, Toronto, Canada; 4 Thoracic Surgery, Department of Surgical Oncology, Nagasaki University, Nagasaki, Japan; 5 Division of Plastic and Reconstructive Surgery, Department of Surgery, Yale New Haven Hospital, Yale School of Medicine, New Haven, CT USA; 6 Institute of Biomaterials and Biomedical Engineering, University of Toronto, Toronto, Canada; 7 Department of Laboratory Medicine and Pathobiology, University of Toronto, Toronto, Canada

**Keywords:** Scaffold, Re-endothelialization, Lung, Transplantation, Vascularization, CD31, Decellularization, Recellularization, Bioreactor, Micro CT imaging

## Abstract

Recellularization of acellular lung scaffolds is a promising technique for the generation of bioengineered lungs. However, at the present time, pre-clinical evaluation of bioengineered lungs shows graft failure likely due to thrombus formation and bleeding from bronchi. This is believed to be due to the impairment of barrier function, incomplete vascular endothelial cell coverage, and limited endothelial cell function. Therefore, efficient recellularization of the vasculature region of the lung scaffold with functional endothelial cells is an important goal. Coating of decellularized tissues, prior to recellularization has shown improved vascular endothelial cell coverage and endothelial cell function. Here, we report the first demonstration of preconditioning decellularized lung vasculature with an anti‑CD31 antibody to enhance re-endothelialization and vascular function in bioengineered lungs. Presence of the anti-CD31 antibody (Ab) coating resulted in enhanced cell adhesion, viability, proliferation and expression of key endothelial cell genes including Claudin-1 (CLDN) and Endothelial Nitric Oxide Synthase (eNOS). Re-endothelialized scaffolds in the anti-CD31 Ab coated group had morphological reconstitution of the pulmonary vasculature and a higher percentage of endothelialized blood vessels. Markedly greater fractions of intravascular Dextran in the anti-CD31 Ab coated group (20.7 ± 2.5% vesus 51.7 ± 2.5%, *p* = 0.0001) demonstrated reduced vascular endothelium leakage. Importantly, scaffolds in the anti-CD31 Ab coated group showed reduced platelet aggregation, measured via staining and quantification for integrin αIIb, when perfused with whole-blood ex vivo (17.7 ± 3.8% vs. 6.7 ± 1.5%, *p* = 0.04); and in vivo following a 30 min-left orthotopic left lung transplant in the mouse (14.7 ± 2.5% vs. 4.3 ± 1.5% in the anti-CD31 group (*p* = 0.004); suggesting reduced thrombus formation. Taken together, our results frame scaffold preconditioning with anti-CD31 Ab as a viable approach towards the generation of vascularized bioengineered lung constructs.

**Supplementary Information:**

The online version contains supplementary material available at 10.1186/s13036-025-00593-x.

## Graphical Abstract

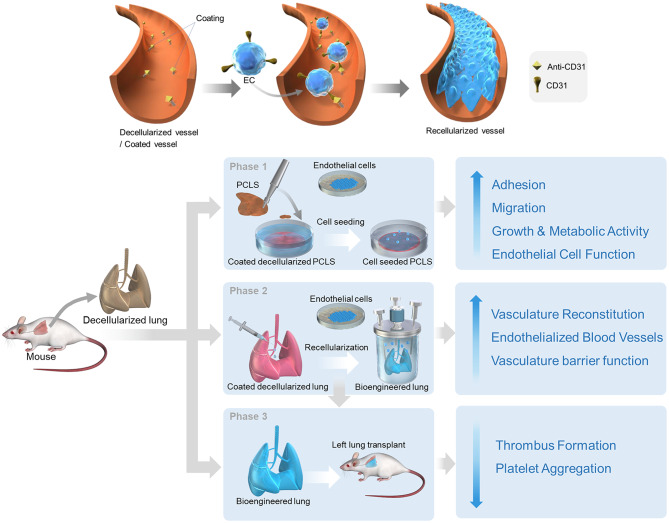

## Introduction

End-stage lung disease is a leading cause of death worldwide, and lung transplantation remains the only curative therapy. It is however, limited by donor shortage and post-transplant complications such as primary graft dysfunction, rejection, chronic lung allograft dysfunction and immunosuppression-related toxicity [1, 2]. Thus, there is a need for alternative solutions to both alleviate the organ shortage and address transplant related limitations. Recellularization of acellular biological scaffolds has emerged as a promising strategy for lung regeneration [3–5]. Decellularized matrices derived from native tissues retain extracellular matrix (ECM) architecture and bioactive cues, including growth factors and structural proteins, that can support endogenous repair and regulate cellular phenotype and function [6, 7].

Pioneering studies extended this concept to whole organs and lungs recellularized with vascular and parenchymal cells achieved partial functional recovery ex vivo and after short-term implantation into preclinical animal models [8, 9]. Since then, decellularization–recellularization approaches have been applied to multiple tissues and organs [8–13]. Despite progress, there are inherent challenges in the field. In the lung, although decellularization protocols and cell sources for recellularization have progressed [14, 15], preclinical transplantation of bioengineered lungs still results in graft failure at least in part due to thrombus formation and airway bleeding [9, 16, 17]. These likely reflect defective vascular-barrier formation, with inadequate endothelial coverage and compromised endothelial function. Recent work has emphasized that preserving and restoring the vascular niche is central to engineered lung performance [18]. Thus, efficient recellularization of the vasculature with functional endothelial cells (ECs) capable of maintaining barrier integrity and resisting thrombosis remains a key objective.

To improve re-endothelialization and EC retention, several groups have functionalized decellularized scaffolds with bioactive proteins or peptides. Specifically, preconditioning with surface coatings have been shown to enhance endothelialization, improve graft patency and reduce thrombosis in engineered vessels, valves and whole-organ scaffolds including liver and kidney [19–25]. We therefore selected the following panel of endothelial-supportive coatings with documented effects on EC adhesion, survival and barrier function on decellularized tissues: Fibronectin, REDV peptide, anti-CD31 antibody, angiopoietin‑1 plus vascular endothelial growth factor (VEGF), and heparin plus gelatin [24–28]. Amongst these candidates, anti-CD31 was of particular interest as CD31 (PECAM‑1) is highly expressed at endothelial cell–cell junctions, contributes to maintenance of vascular-barrier integrity, and can be targeted to promote selective capture and stabilization of ECs on biomaterial surfaces [26].

In this proof-of-concept study, we first screened these coatings on precision-cut lung slices (PCLS) prepared from decellularized lungs [29, 30], assessing endothelial adhesion, viability, metabolic activity, migration and maintenance of phenotype following seeding with a vascular EC line. Based on this screen, we then translated the most promising coating, anti-CD31, to whole lung recellularization in an ex vivo bioreactor and evaluated its impact on vascular thrombogenicity during ex vivo blood perfusion and after short-term in vivo transplantation. We hypothesized that preconditioning the vascular luminal surface of decellularized lungs with anti-CD31 coating would enhance endothelial coverage and vascular-barrier function and thereby reduce thrombosis in bioengineered lungs.

## Materials and methods

### Animal ethics and housing

All animal experiments were approved by the Institutional Animal Care and Use Committee (IACUC) of University Health Network under protocol (approval no. 2787) and were conducted in accordance with institutional and national guidelines. Mice were housed in groups of five adults per cage with unrestricted access to a 5% irradiated chow diet. Environmental conditions were maintained at 21–22 °C with 45–60% relative humidity under a 12-hour light/dark cycle.

### Decellularization of mouse lungs

Decellularization was performed according to the protocol described previously [31–33]. Briefly, C57BL/6 male mice (Jackson Laboratory, Bar Harbor, ME) aged 8 to 12 weeks were euthanized by CO₂ inhalation performed by trained personnel. The euthanasia chamber was gradually filled with CO₂ at a flow rate of approximately 40% of the chamber volume per minute, to minimize distress and reduce the pain associated with carbonic acid irritation of the mucous membranes. Heart-lung blocks were then removed. The main pulmonary artery (PA) and pulmonary vein (PV) were cannulated by a catheter (3-Fr, Instech, PA, USA), and a 22-gauge I.V. catheter (BD, NJ, USA) was inserted into the trachea. The catheters were secured using 4 − 0 silk sutures. The extracted heart-lung block was stored in distilled water (dH₂O) at 4 °C for 1 h followed by perfusion of airways and vasculature with dH₂O. Samples were then stored in 0.1% Triton X100 solution at 4 °C overnight. This was followed by another wash with dH₂O and stored in 2% sodium deoxycholate solution at 4 °C for 24 h. The samples were then washed with dH₂O and stored in sodium chloride solution at room temperature (RT) for 1 h followed by another round ofdH₂O and storage in DNase I (0.1 mg mL⁻¹, DN25-100MG; Thermo Fisher Scientific, MA, USA) solution at RT for another hour. Subsequently, airways and vasculature were washed with PBS and the decellularized lung stored in PBS and 1% antibiotic-antimycotic solution (450-115-EL; Thermo Fisher Scientific, MA, USA) at 4 °C. Quality of decellularization was evaluated by DNA assay and histological evaluation. DNA quantification was done using the Quant-iT PicoGreen dsDNA assay kit (P7589; Thermo Fisher Scientific, MA, USA) as per manufacturer instructions. Briefly, lung tissue samples were weighed and lyophilized, diluted in TE buffer and mixed with the Quant-iT PicoGreen reagent. Fluorescence was measured at 535 nm with excitation at 485 nm, and DNA content was quantified using a standard curve.

### Assessment of mechanical properties

To evaluate the preservation of extracellular matrix (ECM) integrity following decellularization, both mechanical testing and histological analyses were performed on native and decellularized mouse lung scaffolds (n = 3 for each group). Scaffold samples were subjected to uniaxial tensile stress testing on CellScale BioTester 5000 using rectangular strips (approximately 0.6 mm × 1 mm × 4.5 mm) excised from the left lobe of each lung. Each strip was mounted on the uniaxial testing apparatus along its long axis and stretched under a controlled rate (0.2 mm per second) until failure to generate stress–strain curves, from which elastic behavior and stiffness parameters were derived using MATLAB software.

### Preparation of precision cut lung slices (PCLS)

PCLS was made according to the previously described techniques [34]. Briefly, the decellularized mouse whole lungs were cleared via perfusion of 3 mL of sterile phosphate-buffered saline (PBS; WISENT INC. QC, Canada) through the main pulmonary artery. Immediately after, the lungs were filled via tracheal perfusion with 2 mL of 2% low melting point agarose (A9414; Sigma-Aldrich, MO, USA) dissolved in PBS. The whole lung was placed on ice for 15 min before the lungs were extracted and placed in PBS with 1% antibiotics/antimycotics. Upon solidification, the lobes were sliced using a vibrating microtome (Leica Biosystems Inc. Nussloch, Germany), yielding PCLS of uniform thickness of 250 μm. PCLS were punched to 5 mm diameter discs for study.

### Preparation and optimization of PCLS surface coatings

Decellularized precision-cut lung slice (PCLS) discs were coated with bioactive molecules using established protocols. For heparin-gelatin coating, discs were incubated for 1 h in 0.1% gelatin (ES006; Sigma-Aldrich, MO, USA) containing 0.1% heparin sodium salt (H4784; Sigma-Aldrich. MO, USA) [27]. For REDV peptide coating, discs were first activated with a mixed solution of 1-(3-dimethylaminopropyl)-3-ethylcarbodiimide hydrochloride (EDC) (AAA1080706; Thermo Scientific Chemicals, MA, USA), N-hydroxysuccinimide (NHS) (24500; Thermo Scientific Chemicals, MA, USA), and ulvan (ULV102; Elicityl, Grenoble, France) for 4 h at 37 °C, followed by PBS washing and incubation with REDV peptide (F4215-98 K; United States Biological, MA, USA) at 0.2 mg mL^− 1^ for 4 h at 37 °C [24, 35]. Ulvan served as a linker for peptide attachment. For antibody and protein coatings, discs were incubated overnight at 4 °C with either 50 µg mL^− 1^ anti-CD31 antibody (ab222783; Abcam, Cambridge, UK), 50 µg mL^− 1^ fibronectin (ab45688; Abcam, Cambridge, UK), or 1 µg mL^− 1^ each of angiopoietin-1 (50300-M07H-100; Sino Biological Inc., Beijing, China) and VEGF (CLY200-34; Cedarlane, ON, Canada) [25, 26, 28]. Optimal concentrations for each coating agent were determined by dose-response adhesion assays using ranges reported in the literature: anti-CD31 and fibronectin (6.25–100 µg mL^− 1^), angiopoietin-1 + VEGF (0.25–4 µg mL^− 1^), heparin (0.025–0.4%), and REDV peptide (0.05–0.8%). For all subsequent experiments, the optimized concentration and incubation time for each reagent were kept constant across PCLS and whole-lung scaffolds to minimize variability. To assess retention of anti-CD31 coating, discs were incubated overnight at 4 °C with anti-CD31 antibody at 12.5, 25, 50, or 100 µg mL^− 1^ in 96 well plates, stained with donkey anti-rabbit IgG AlexaFluor™ 488 (A-21206; Thermo Fisher Scientific, MA, USA) and Hoechst 33,342, imaged using fluorescence microscopy (Axioscan 7; Zeiss, Oberkochen, Germany), and quantified using ImageJ.

### Cell culture

In this study, mouse endothelial cells (C166) (ATCC, Manassas, VA) were predominantly used. C166 cells were cultured in high-glucose content Dulbecco’s Modified Eagle’s Medium (DMEM) (12430054; Thermo Fisher Scientific, MA, USA) containing 10% fetal bovine serum (12483020; Thermo Fisher Scientific, MA, USA), and 1% antibiotic-antimycotics (Thermo Fisher Scientific, MA, USA). Cells were stored in a standard incubator (95% air and 5% CO₂ at 37 °C). Every 2 to 3 days, the cell culture media was changed, and cells were passaged once they reached confluence. Endothelial cell growth medium (EGM) (Lonza Bioscience, MD, USA) was used to culture human umbilical vein endothelial cells (HUVEC) (ATCC, Manassas, VA). Cells were stored in a standard incubator (95% air and 5%CO₂ at 37 °C). Every 1 to 2 days, the cell culture media was changed, and cells were passaged after reaching confluence.

### In vitro cell attachment assay

To evaluate the ability of the coating agents to support the attachment of C166 cells, PCLS were prepared after decellularization, the 5 mm discs were treated with each coating agent. Discs of PCLS treated with PBS were used as negative controls. After the coating process, 100 µL of cell suspension containing 5 × 10^3^ C166 cells or 5 × 10^3^ HUVECs were applied to the disc surfaces. The 96-well plate was subsequently incubated at 37 °C in 95% air / 5% CO₂ for 1 day to facilitate cell attachment. The substrates were washed with PBS and stained with Hoechst 33,342 (H3570; Thermo Fischer Scientific, MA, USA) according to the manufacturer’s instructions. Each disc was imaged using a confocal microscope (Nikon, Tokyo, Japan). Quantification was performed using the ImageJ software [36].

### In vitro cell metabolic activity assay

After the coating process, a 100 µL of cell suspension containing 5 × 10^3^ C166 cells or 5 × 10^3^ HUVECs was applied to the disc surfaces in 96-well plates. The plates were subsequently incubated at 37 °C in 95% air / 5% CO₂ for 1, 3, or 5 days to allow cell proliferation. Before measurement, samples were washed with PBS, incubated with 100 µL of fresh culture medium containing 10 µL Cell Counting Kit-8 (CCK-8) (96992; Sigma-Aldrich, MO, USA) solution for 2 h, transferred into a new 96-well plate, and incubated at 37 °C. The medium was transferred to a new 96-well plate and the relative cell numbers were determined by measuring the optical density at 450 nm in each well using a microplate reader (Cytation 5 Cell Imaging Multi-Mode Reader BioTek, Agilent Technologies, CA, USA).

### Endothelial cell (EC) migration assay

To check the ability of uncoated and precoated decellularized PCLS to stimulate migration of ECs, a transwell chamber assay was performed using 8 μm pore size Cell Migration Assay Kit (ab235673; Abcam, CA, USA) as previously described [27, 37]. Discs of decellularized PCLS (5 mm in diameter) either precoated with each coating agents or PBS were placed at the bottom of the lower chamber of a 96-well plate. In this setup, no exogenous chemokines were added. Instead, decellularized PCLS in the lower chamber release extracellular matrix-derived soluble factors and matrix-associated molecules that establish a scaffold-derived chemotactic gradient across the porous membrane [38–40]. Wells without PCLS were used as a control group to exclude the effect of gravity on cell migration. Then, 150 µL DMEM was added into the lower chamber. Suspensions of 50 µL of 1 × 10^6^ cells/mL C166 cells labeled with Hoechst 33,342 were seeded in the upper chamber. The plate was incubated at 37 °C and 5%CO₂ for 8 h to allow cells to migrate. After 8 h incubation, cells that remained on the upper side of the membrane were removed with a cotton swab. The transwell membrane was rinsed with PBS, and cell migration into the lower chamber was assessed by measuring the fluorescence in the lower compartment in 5 random microscopic fields using a confocal microscope (Nikon, Tokyo, Japan). Quantification was done using ImageJ software.

### Whole lung scaffold coating

For REDV coating, a mixed solution (EDC, NHS, and ulvan), was injected and the lungs were incubated for 4 h at 37 °C. Subsequently, the lungs were washed with PBS, followed by the injection of REDV solution (0.2 mg mL⁻¹) into the pulmonary artery and vein. The lungs were then incubated for 4 h at 37 °C. For anti-CD31 Ab and fibronectin coatings, either 50 µg mL⁻¹ anti-CD31 Ab or 50 µg mL⁻¹ fibronectin were injected into the pulmonary artery and vein and incubated overnight at 4 °C. After incubation with each coating agent, the lungs were washed with PBS. For HUVEC re-endothelialization, a 25 µg mL⁻¹ anti-CD31 Ab solution was injected into the pulmonary artery and incubated overnight at 4 °C. ​These incubation times were based on previously published decellularized organ and vascular scaffold studies using the same reagents [24–28] and were applied as fixed conditions across all whole-lung experiments for each respective coating.

### Re-endothelialization of mouse whole lung scaffolds

Our established mouse-scale perfusion-based bioreactor was set up as previously described [9, 37]. For gravity-driven cell delivery, a cell reservoir that contained 5 million C166 endothelial cells or HUVECs was placed 30 cm above the organ chamber in which a decellularized lung was connected through the PA cannula. The injection rate was 1 mL min⁻¹. After cell injection, the organ chamber was placed in a CO_2_ incubator for one hour. Then the chamber was connected to the media circuit. Media flow was generated by a Masterlfex L/S Precision Modular Drivers with Bench Top Controller (Cole-Parmer, IL, USA) at a rate of 0.3 mL min⁻¹ for three days. Following the 3-day culture period in the bioreactor, re-endothelialized whole lungs were prepared for histological evaluation and immunostaining and gene expression analysis. Samples to be used for histology were rinsed in PBS and fixed by immersion in 10% neutral buffered formalin for 24 h, embedded in paraffin blocks, and sectioned at 5 μm for subsequent histological and immunofluorescence analyses. For gene-expression analysis, pieces of parenchyma were dissected from several lobes of each lung, pooled into a single sample per lung, snap-frozen, and stored at − 80 °C until RNA extraction. Tissue was not pooled between different animals.

To assess the retention of anti-CD31 antibody (Recombinant Anti-CD31 antibody [EPR17260-263], Abcam, Cambridge, UK) coating post whole mouse lung C166 re-endothelialization, the samples were fixed with 10% formalin solution for 1 day then embedded in paraffin blocks and sectioned at 5 μm. Sections were then deparaffinized before undergoing antigen retrieval via Ninja pressure cooker at 120 °C for 15 min. Next, the samples were treated with ITFX Signal Enhancer (I36933, Thermo Fischer Scientific, MA, USA) and 1% bovine serum albumin blocking solution (15260037, Thermo Fischer Scientific, MA, USA) followed by application of donkey anti-rabbit IgG secondary antibody AlexaFluor™ 488 (A-21206, Thermo Fischer Scientific, MA, USA). After mounting with ProLong Gold antifade mounting medium, the slides were imaged via confocal microscopy (A1R point scanning confocal microscope, Nikon, Tokyo, Japan).

### Terminal deoxynucleotidyl transferase dUTP Nick end labeling (TUNEL) staining

TUNEL staining was conducted according to the manufacturer instructions (In Situ Cell Death Detection Kit; 12156792910; Roche Diagnostics, Germany). Briefly, tissue slices were deparaffinized, antigen repaired with Proteinase K (ab64220; Abcam, Cambridge, UK) working solution and blocked with 5% Donkey Serum (D9663; Sigma-Aldrich, MO, USA) at room temperature (RT) for 1 h. Immunofluorescence was performed using TUNEL reaction mixture for 60 min at 37 °C. After washing with PBS three times, the nuclei were stained by diamidino-2-phenylindole (DAPI; 10236276001; Sigma-Aldrich, MO, USA. Finally, fluorescence images were observed with a fluorescence microscope. To quantify the numbers of apoptotic cells, images of 3 randomly chosen fields/slides were taken for each repopulated lung and TUNEL-positive cells were manually counted.

### Immunostaining

Samples were fixed in 10% neutral buffered formalin for 24 h, embedded in paraffin blocks, and section at 5 μm thickness. Three sections were prepared per lung. Hematoxylin (HHS32; Sigma-Aldrich, MO, USA) and eosin (71304; VWR, Canada) staining was performed according to established protocols [33, 41]. For extracellular matrix evaluation, Masson’s Trichrome (StatLab, KTMTRPT EA), Verhoeff-Van Gieson (ab150667; Abcam) and Alcian Blue (ab150662; Abcam) were used to visualize collagen, elastin, and sulfated glycosaminoglycans (sGAGs), respectively. Stained slides were imaged using Leica Aperio CS2, and quantitative analyses were performed to compare ECM composition and tissue architecture between groups.

For immunofluorescence, sections were deparaffinized, treated with a blocking solution (Protein Block Serum-Free; X090930-2; Agilent Dako, CA, USA), and incubated overnight at 4 °C with primary antibodies against laminin (1:100, 4H8-2; Sigma-Aldrich), VE-cadherin (1:200, BV13; BioLegend), ZO-1 (1:100, ab216880; Abcam), Integrin αIIb from pig (1:200, orb4832; Biorbyt) and mouse (1:100, ab134131; Abcam), and caspase-3 (1:50, 700182; Thermo Fisher Scientific). Secondary antibodies included AlexaFluor 647 donkey anti-rabbit IgG for laminin, ZO-1, and caspase-3 (1:500, A21244; Thermo Fisher Scientific), AlexaFluor 546 goat anti-rat IgG for VE-cadherin (1:500, A11081; Thermo Fisher Scientific), and AlexaFluor 546 goat anti-rabbit IgG for Integrin αIIb (1:500, A10040; Thermo Fisher Scientific). Sections were mounted with ProLong Gold antifade mounting medium containing DAPI (P36930; Thermo Fisher Scientific). Imaging was performed using a whole-slide scanner (AxioScan™; Zeiss, Germany), and quantification of ZO-1 and Integrin αIIb-positive areas was carried out using ImageJ software, expressed as a percentage of total lung parenchyma. Data represents means from three slides per lung scaffold, with three randomized fields per slide.

For Ki67 immunohistochemistry, a heat-induced antigen retrieval procedure was performed using a citrate buffer solution (Antigen Retrieval Buffer 100 x pH 6.0, ab93678; Abcam) in a pressure cooker for 15 min. Sections were then incubated with 3% H_2_O_2_ for 10 min at room temperature (RT) to block peroxidase, followed by 10 min at RT with Dako protein block (X0909; Dako) to block endogenous proteins. Samples were then incubated for 30 min with the primary antibody – rabbit mAb Ki67 (MA5-14520; Thermo Fisher Scientific). Antibody reactions were then visualized using Vector Impress HRP Horse anti-rabbit IgG plus polymer and ImmPACT DAB with horseradish detection system (SK-4105; Vector Lab). Stained slides were imaged using Leica Aperio CS2.

###  X-ray micro-computed tomographic (µCT) lung preparation, imaging and vasculature analysis

To improve the contrast of the pulmonary circuits for X-ray microcomputed tomography (µCT) imaging, a liquid contrast agent (Microfil MV-117, Flow Tech Inc, CT, USA) was injected into the lungs. The ratio of the contrast agent to the diluent to the curing agent was set to 8:10:1. A syringe pump (NE-1000, New Era Pump Systems Inc, NY, USA) was used to deliver 1 mL of the contrast agent solution into the vasculature of the lungs at a steady flow rate of 0.1 mL min⁻¹ via the pulmonary artery.

Micro-CT imaging was performed on contrast agent-injected mouse lungs to acquire three-dimensional reconstructions of the pulmonary vasculature. To prevent movement, contamination, and dehydration during scanning, lungs were placed in falcon tubes surrounded by PBS-soaked cotton. Scans were conducted at 110 kV and 140 µA with a rotational step of 0.167°, exposure time of 0.4 s, and averaging of two frames, generating 2160 projections at a voxel size of 8–12 μm per pixel. Images were initially reconstructed as 16-bit files and converted to 8-bit to reduce memory load and processing time. Post-reconstruction, Gaussian blur filtering was applied to minimize intensity variance and prevent segmentation artifacts such as non-physical holes, followed by a vesselness filter to enhance contrast between vessels and void spaces, as previously described [42, 43]. Segmentation was performed using Otsu’s global thresholding to binarize images, isolating the largest connected voxel cluster representing the continuous vascular path created by the contrast agent. Vasculature morphology, including vessel diameter, length, and connectivity, was quantified using the Vessel Network Extraction algorithm [42]. Vessel length was calculated by summing Euclidean distances along centerlines, and diameters were estimated using a fast-marching method assuming cylindrical geometry. Gradient and diameter-length filters were applied to remove spurious branches while preserving network connectivity, enabling accurate volumetric analysis of native, decellularized, and re-endothelialized lungs.

### FITC-conjugate dextran perfusion assay

According to previous studies [15, 26, 44] the permeability of re-endothelialized vessels in engineered tissues was assessed by perfusion of 500 kDa FITC-dextran (FD500S, Sigma Aldrich, MO, USA) via the pulmonary artery. For gravity-driven injection, 10 mL of FITC-Dextran (0.2 mg mL⁻¹) was administered from 15 cm above the lung. Dextran in the drained fluid from the pulmonary veins were considered as intravascular dextran, whereas that from the periphery and trachea was referred to as extravascular dextran. The amount of total dextran was measured by fluorescence intensity (Cytation 5 Cell Imaging Multi-Mode Reader BioTek, Agilent Technologies, CA, USA) (absorbance at 490 nm) multiplied with volume of drained fluid.

### Ex vivo whole-blood perfusion

To assess scaffold thrombogenicity and the efficiency of endothelialization, precoated or uncoated reendothelialized scaffolds were retrieved after 3 days in the bioreactor, followed by 24-hour perfusion with freshly harvested porcine blood mixed (1:1) with perfusion medium (DMEM containing 10% fetal bovine serum and 1% antibiotic-antimycotics). Note that heparin (10,000 units) was injected intravenously into the male Yorkshire pigs (28–33 kg) prior to blood being taken. The chamber was connected to the media circuit. Media flow was generated at a rate of 0.3 mL min⁻¹ at 37 °C. Non-seeded decellularized scaffolds were used as controls. After perfusing with blood, scaffolds were rinsed in PBS and fixed by immersion in 10% neutral buffered formalin for 24 h, then processed routinely, embedded in paraffin blocks, and sectioned at 5 μm for histological and immunofluorescence analyses. For gene-expression analysis, pieces of parenchyma were dissected from several lobes of each lung, pooled into a single sample per lung, snap-frozen, and stored at − 80 °C. Samples were then subjected to RNA extraction using TRIzol and column purification (RNeasy Mini Kit, Qiagen), followed by cDNA synthesis and SYBR Green–based quantitative real-time PCR with GAPDH as the housekeeping gene, as detailed in the Quantitative PCR section. For quantification of platelet adhesion, integrin αIIb platelet fluorescence intensity was obtained from randomly selected images. Blood samples were collected at different time points for platelet cell counting using blood smear.Note that fresh porcine blood was obtained from animals enrolled in a separate Institutional Animal Care and Use Committee (IACUC)–approved protocol at the University Health Network (AUP 6474.14). No additional animals were euthanized solely for the purpose of this study.

### Orthotopic lung transplantation of bioengineered lung

Orthotopic left lung transplantation was performed using male C57BL/6J mice (8–12 weeks old) as both donors and recipients, as previously described using a cuffed technique [45, 46] with the modifications below. All transplantation procedures were conducted under the same Institutional Animal Care and Use Committee (IACUC) approval as the other mouse experiments in this study (protocol no. 2787, University Health Network). C uffs made with 24G, 22G, and 20G angiocatheters were attached to the bioengineered lung’s left pulmonary artery, vein, and bronchus, respectively. Recipient mice were anesthetized with an intraperitoneal injection of a mixture of ketamine (0.1 mg kg⁻¹), xylazine (0.01 mg kg⁻¹), and 2% of isoflurane during surgery. After 30 min of implantation, euthanization was performed, and the implanted left lung scaffolds were retrieved. Tissue samples were collected for histology and RNA extraction.

### Quantitative PCR

Tissues were disrupted in TRIzol (15596026; Thermo Fisher Scientific) using TissueLyserII (Qiagen, Venlo, Netherlands) according to the manufacturer’s instructions. Total RNA was purified using the RNeasy mini kit (74104; Qiagen) according to manufacturer’s instructions. The amount of RNA and 260/280 ratios were measured using NanoDrop1000 (Thermo Fisher Scientific). To prepare cDNA, total RNA was used for reverse transcription using the QuantiTect Reverse Transcription Kit (205311; Qiagen). Quantitative PCR was performed using Universal SYBR Green Supermix (1725274; Bio-Rad Laboratories, CA, USA) with CFX384 (Bio-Rad, CA, USA). All primer sets used for this study are listed in Supplemental Table 1. *GAPDH* was used as a housekeeping gene, and each group was analyzed using the ΔΔCt method.

### Statistical analysis

All data are reported as mean ± standard deviation (SD). All statistical analyses were performed using GraphPad Prism software (version 10.1.0; GraphPad Software). In all experiments, “n” refers to biological replicates, defined as independent samples (e.g., independent decellularized/re-endothelialized lungs, independent PCLS preparations, or independent cell culture experiments performed on different days). Technical replicates (such as multiple imaging fields from the same lung/PCLS or multiple qRT-PCR wells from the same RNA preparation) were averaged to yield a single value per biological replicate before performing statistical tests. For whole lung scaffold experiments, we used *n* = 3 biological replicates per group, where each n corresponds to an independent decellularized/re-endothelialized lung (or lung scaffold) processed and analyzed separately. Within each biological replicate, measurements were averaged from multiple technical replicates (e.g., 3–5 imaging fields per lung and 3 qPCR wells per condition).

For all multi-group comparisons, we checked normality (Shapiro–Wilk test) and homogeneity of variance (Brown–Forsythe or Levene test). When the data satisfied these assumptions, a one-way ANOVA followed by Tukey’s post hoc test for pairwise comparisons was used. Experiments with two groups and a single independent variable were assessed by two-tailed student’s t-test. Experiments with three or more groups and a single independent variable were assessed by one-way ANOVA with a Tukey’s test for multiple comparisons. Values of *p* < 0.05 were considered significant. When normality or equal-variance assumptions were not met, non-parametric tests (Kruskal–Wallis with Dunn’s post hoc test) were utilized.

## Results

### Coating with bioactive factors increases adhesion, viability and proliferation of mouse vascular ECs in precision cut lung slices (PCLS)

We produced decellularized mouse lungs, which displayed a complete absence of cells, accompanied by a significant reduction in DNA content compared to native lungs (Supplemental Fig. 1). Characterization of decellularized mouse lung scaffolds revealed that the decellularized scaffolds had reproducible mechanical and compositional characteristics that were similar to native lungs (Supplementary Fig. 1), ensuring physiologically relevant conditions for subsequent re-endothelialized experiments. Specifically, we evaluated collagen, elastin, sulfated glycosaminoglycans (Supplementary Fig. 2A) and the elastic behaviour of the decellularized scaffold (Supplementary Fig. 2B).

PCLS were then generated from the decellularized lungs by filling lung tissue with agarose to provide stability and then sectioned to generate intact tissue slices that can be maintained in standard tissue culture conditions. Thus, we maintained a 3D culture platform with preserved native architecture [47, 48]. PCLS were then coated with bioactive factors (heparin + gelatin, fibronectin, angiopoietin-1 + VEGF, REDV, anti-CD31 Ab), or left uncoated. The starting concentration of each factor was selected based on previously reported studies [24–28] and optimized via dose-response adhesion assays (Supplemental Fig. 3). Final concentrations used were: 0.1% for heparin + gelatin, 50 µg mL⁻¹ for fibronectin, 1 µg mL⁻¹ each for angiopoietin-1 + VEGF, 0.2 mg mL⁻¹ for REDV, and 50 µg mL⁻¹ for anti-CD31 Ab. Retainment of the anti-CD31 coating on the scaffold was confirmed via immunofluorescence staining of the antibody (Supplementary Fig. 3A).

**Fig. 1:**
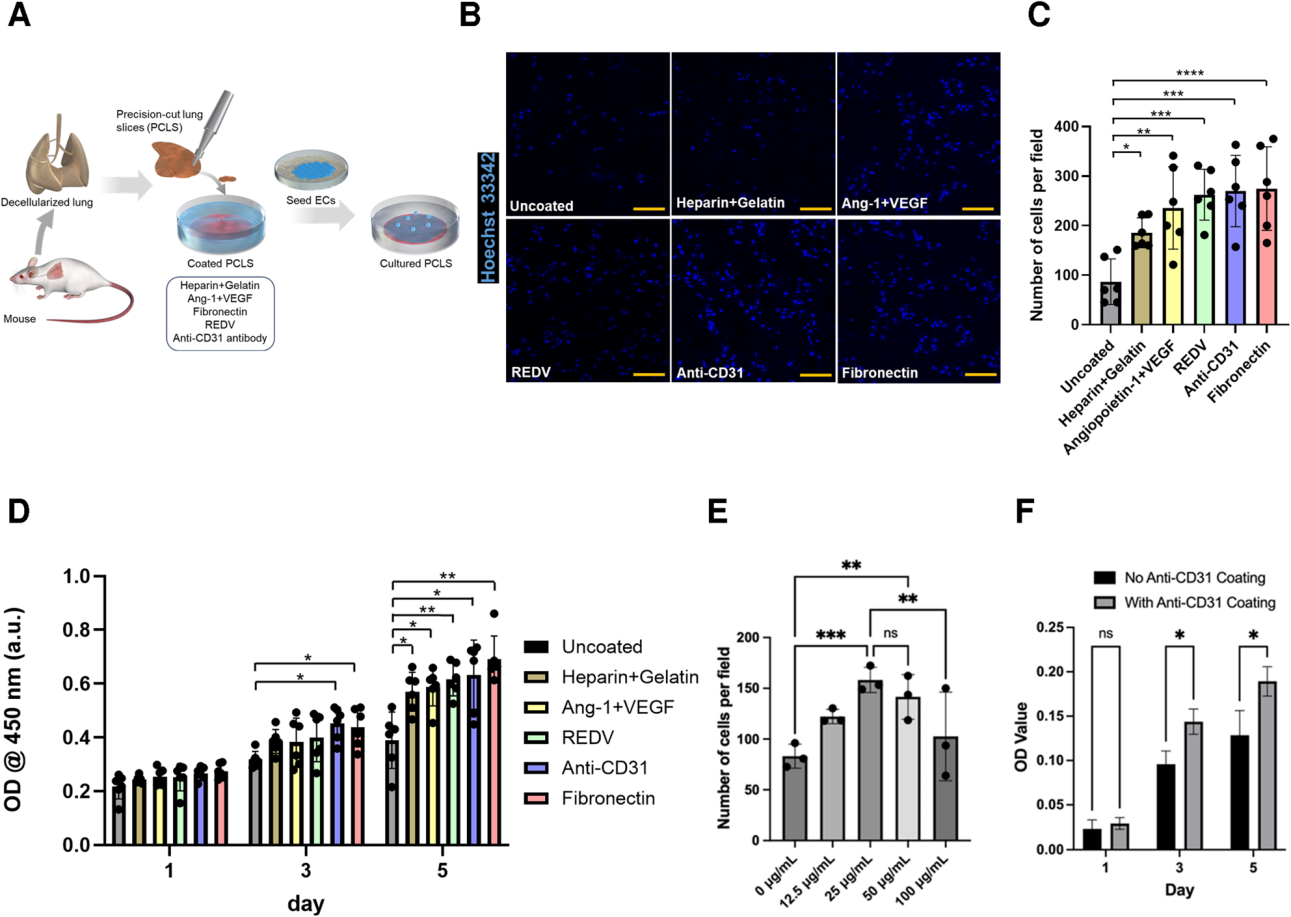
Effect of coating agents on the attachment and growth of Endothelial cells (ECs) on precision cut lung slices (PCLS). (**A**) Schematic diagram describes the experimental design of each assay using decellularized PCLS. (**B**, **C**) Coating of decellularized scaffolds enhance the attachment of Hoechst 33,342-labeled (blue) mC166 cells on PCLS compared to the uncoated group. Each group *n* = 6. (**D**) Quantification of EC cell metabolic activity using cell growth assay using Cell Counting Kit-8 (CCK-8) revealed that precoating of PCLS increases ECs growth and metabolic activity (Optical Density (OD) measured at Day 1, 3 and 5) compared to ECs cultured on uncoated PCLS. Each group *n* = 6. (**E**) Quantification of the attachment of Hoechst-labeled HUVECs on anti-CD31 coated PCLS coated with 12.5, 25, 50, or 100 µg/mL anti-CD31 Ab. Results represent the mean of measurements taken from 3 PCLS seeded with HUVEC cells from 3 independent experiments. Each group *n* = 3. (**F**) Quantification of HUVEC cell metabolic activity using cell growth assay using Cell Counting Kit-8 (CCK-8) revealed that precoating of PCLS increases ECs growth and metabolic activity (Optical Density (OD) measured at Day 1, 3 and 5) compared to ECs cultured on uncoated PCLS. Each group *n* = 3. Scale bar = 200 μm. The results represent the mean of measurements taken from 3 technical replicates. Each number represents a biological replicate (independent experiment). M ean ± SD, *p < 0.05, **p < 0.01, ***p < 0.001, ****p < 0.0001

Mouse vascular endothelial cells (C166) were then seeded onto the coated PCLS and subjected to functional assays (Fig. 1A).

After 24 h, the cells adhering to the PCLS were stained with Hoechst 33,342, and adhered cells were evaluated using confocal microscopy (Fig. 1B). The number of adherent cells was greater on all of the coated scaffold groups when compared to that of the uncoated group. In particular, the REDV, fibronectin and anti-CD31 coated groups demonstrated the highest number of cells attached (Fig. 1C).

C166 cell growth was quantified over 5 days via assessment of metabolic activity on days 1, 3, and 5 (Fig. 1D). On day 1, no significant differences were noted among the groups. However, on day 3, the anti-CD31 Ab and fibronectin groups, and on day 5, all coated groups displayed significantly higher metabolic activity compared to that of the uncoated group. To determine the generalizability of the findings to human cells, we evaluated the effect of anti-CD31 coating on human umbilical vein endothelial cells (HUVECs). Our data shows that anti-CD31 coating of acellular mouse lung scaffolds results in a significantly higher number of attached HUVECs on the precision cut lung slices (PCLS) with the cells showing higher metabolic activity over 3 and 5 days in culture (Fig. 1E and F).

### Coating with bioactive factors enhances the migration capacity of mouse vascular ECs on PCLS and increases expression of genes associated with endothelial cell function

**Fig. 2:**
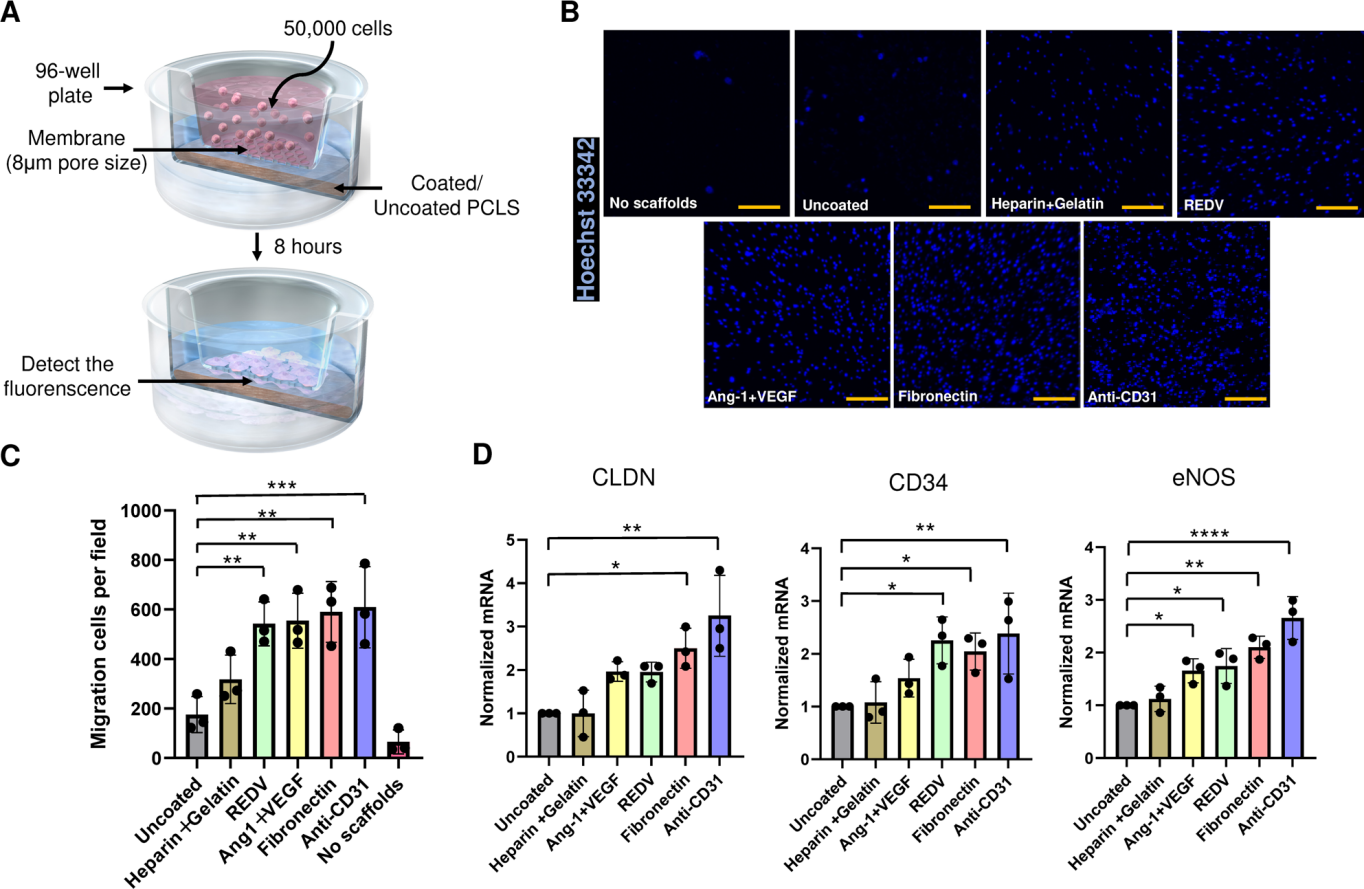
Effect of coating agents on migration and gene expression of ECs cultured on PCLS. (**A**) Schematic diagram describes the experimental design of the EC migration assay using the decellularized PCLS. (**B**, **C**) Coating enhances the migration of Hoechst-labeled cell towards the PCLS compared to the uncoated group. (**D**) In a separate experiment, quantification of *CLDN*, *CD34*, and *eNOS* gene expression was performed by quantitative real-time RT-PCR in C166 ECs seeded on PCLS for 24 h, showing increased gene expression in ECs grown within the coated PCLS compared to uncoated conditions. The results represent the mean of measurements taken from 3 technical replicates. Each number represents a biological replicate (independent experiment). Scale bar = 200 μm. Each group *n* = 3, mean ± SD, *p < 0.05, *p < 0.05, **p < 0.01, ***p < 0.001, ****p < 0.0001

The migratory response of C166 cells toward coated PCLS was assessed using a Transwell assay (Fig. 2A). In this assay, decellularized PCLS placed in the lower chamber release extracellular matrix–derived factors that create a migration-promoting gradient across the membrane in the absence of added chemokines. Following an 8-hour incubation, the number of cells that migrated underneath the membrane was quantified using confocal microscopy (Fig. 2B). Notably, all coated groups, except the heparin + gelatin group, exhibited significantly enhanced cell migration ability compared to that of the uncoated group (Fig. 2C).

In a separate experiment, to evaluate whether bioactive coatings impacted the expression of genes related to vascular endothelial function, C166 ECs were cultured on PCLS for 24 h, after which the expression of genes associated with endothelial cell function was evaluated (Fig. 2D). Specifically, we examined the expression of claudin (*CLDN*), involved in maintaining barrier integrity, as well as *CD34* and endothelial nitric oxide synthase (*eNOS*), which are associated with intercellular interactions and angiogenesis.

Both the anti-CD31 antibody coating and the fibronectin coating group significantly increased all three genes relative to uncoated controls. REDV significantly increased CD34 and eNOS but did not change CLDN; and angiopoietin-1 + VEGF significantly upregulated eNOS only. In summary, these findings suggest that the utilization of specific coating agents enhances the function of seeded vascular endothelial cells.

### Pretreatment of acellular whole lung scaffolds with bioactive factors results in enhanced re-endothelialization

While the selected panel of coating agents all induced a positive response in the endothelial cells on our PCLS platform, for re-endothelialization of whole lung scaffolds, we selected the top three coating agents, namely REDV, fibronectin, and anti-CD31 Ab.

These were selected based on their ability to produce a statistically significant improvement (*p* < 0.05) over uncoated scaffolds in cell adhesion and metabolic activity, together with enhancement of at least one additional functional parameter (migration and/or upregulation of CLDN, CD34, and eNOS).

**Fig. 3:**
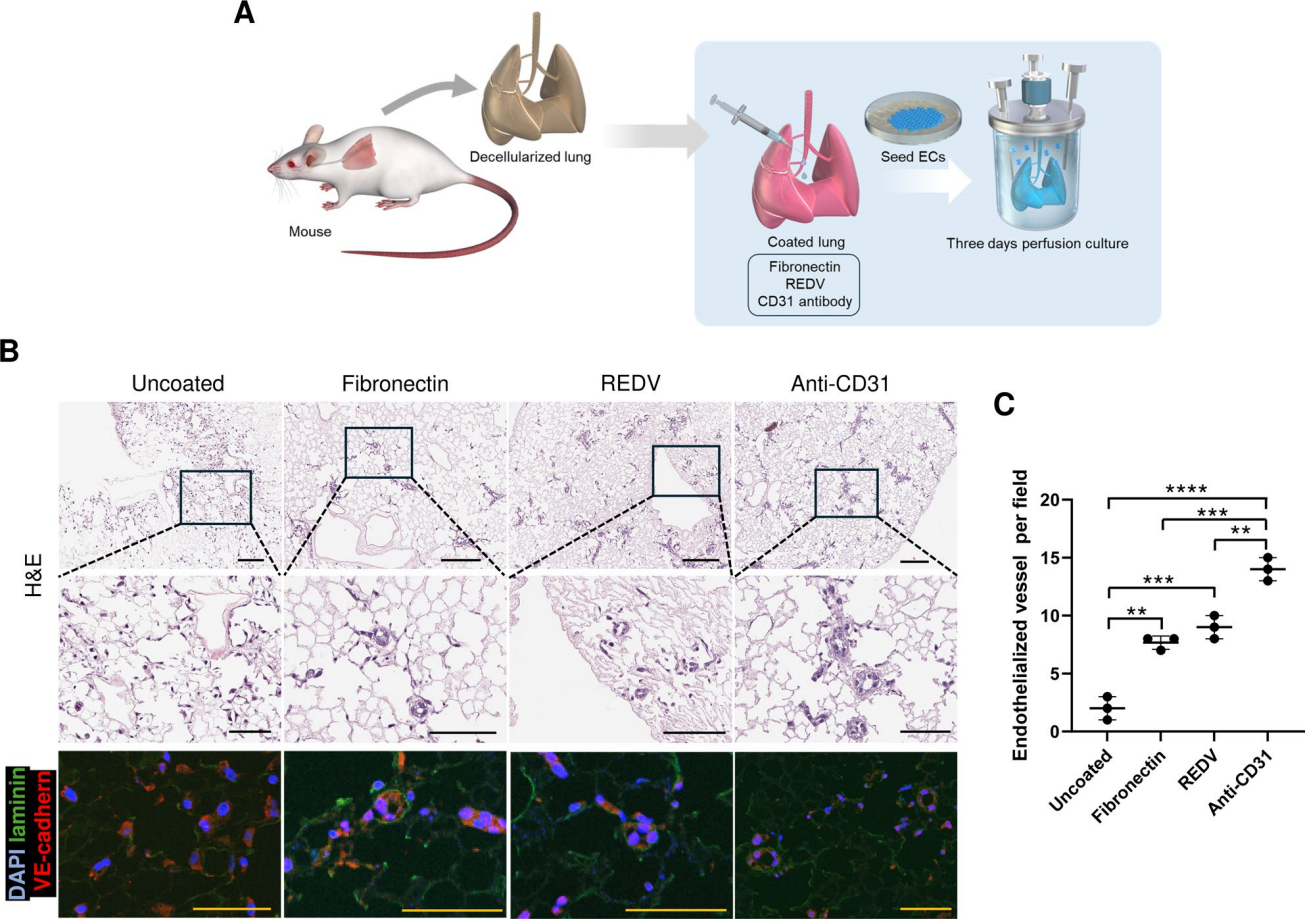
Re-endothelialization of decellularized mouse lung scaffolds. (**A**) Schematic diagram illustrating the strategy for re-endothelialization of the decellularized mouse lung scaffolds with C166 cells. (**B**) Hematoxylin and eosin (H&E) stained images and immunofluorescence images with 4’,6-diamidino-2-phenylindole (DAPI) (blue), laminin (green), and VE-cadherin (red) showed the cells were found to be randomly localized in the uncoated group, whereas in the coated groups, the cells were clearly lining blood vessels. Scale bar = 200 μm (top), 100 μm (middle), 100 μm (bottom). (**C**) Quantification of endothelial vessels per fields in each group. The results represent the mean of three images taken from each recellularized lung. Each number represents a biological replicate (independent whole lung scaffold). Each group *n* = 3, mean ± SD, **p < 0.01, ***p < 0.001, ****p < 0.0001

For whole lung re-endothelialization, the pulmonary blood vessels were coated with either REDV, fibronectin, or anti-CD31 antibody immediately following decellularization. Subsequently, C166 ECs were seeded through the pulmonary artery using gravity cell-seeding methods, and perfusion culture in a bioreactor system [45] was carried out for three days to capture early initiation of endothelial cell-matrix interactions (Fig. 3A). Blood vessel recellularization was evaluated for each coated and uncoated group by hematoxylin and eosin staining, and immunostaining using anti-laminin and VE-cadherin antibodies. The cells were found to be randomly localized in the scaffold in the uncoated group, whereas in the coated groups, seeded endothelial cells were clearly lining blood vessels (Fig. 3B). Quantitative analysis indicated a significant increase in the number of re-endothelialized blood vessels in all coated groups compared to that in the uncoated group, with the anti-CD31 Ab group displaying the highest number of re-endothelialized blood vessels (Fig. 3C). These results demonstrate that pre-coating of whole lung scaffolds before re-endothelialization significantly enhances the efficiency of vascular re-endothelialization by endothelial cells.

### Assessment of vascular endothelial cell viability, proliferation, and expression of endothelial function-related genes

**Fig. 4:**
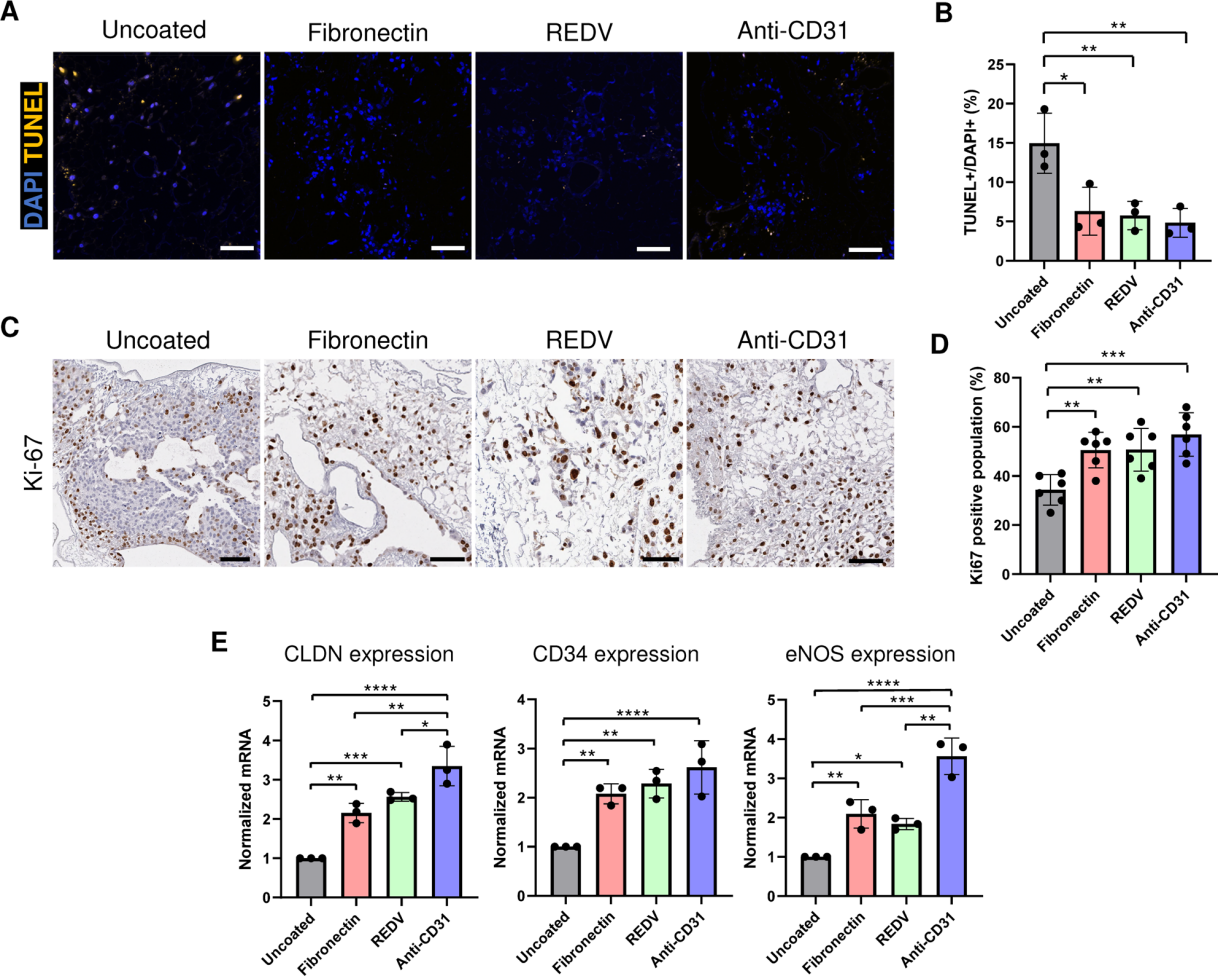
Characterization of re-endothelialized lung scaffold after precoating with fibronectin, REDV or anti CD31 Ab coating agents. (**A**, **B**) Confocal microscopy images of Terminal deoxynucleotidyl transferase (TdT)-mediated dUTP nick end labelling (TUNEL) stained cells (TUNEL (yellow), DAPI (blue)) showing fewer numbers of apoptotic cells in scaffolds coated groups compared to uncoated groups. Each group *n* = 3. (**C**, **D**) Ki-67 staining showed that ECs grown within the coated scaffolds were proliferative. Each group *n* = 6. (**E**) Quantification of *CLDN*, *CD34*, and *eNOS* genes as measured by quantitative real-time RT PCR showing increased levels of expression in ECs grown within the coated scaffolds compared to uncoated ones. The results represent the mean of measurements taken from 3 technical replicates. Each number represents a biological replicate (independent whole lung scaffold). Scale bar = 100 μm. Each group *n* = 3, mean ± SD, *p < 0.05, **p < 0.01, ***p < 0.001, ****p < 0.0001

Subsequently, we examined the viability and proliferation of seeded endothelial cells in the bioengineered lungs. To assess cell viability in coated and uncoated groups, we conducted a Terminal deoxynucleotidyl transferase (TdT)-mediated dUTP nick end labelling (TUNEL) assay (Fig. 4A). We noted a decrease in the percentage of apoptotic cells in all coated groups compared to that of the uncoated group (Fig. 4B). Reduction of apoptosis in the anti-CD31 coated group was also confirmed via Caspase 3 staining (Supplementary Fig. 5). Proliferation was evaluated using immunostaining for Ki-67 (Fig. 4C), which indicated a higher number of Ki-67-positive cells in all coated groups compared to that in the uncoated group (Fig. 4D). Moreover, the expression levels of the three genes (*CLDN*, *CD34*, and *eNOS*) were notably higher in all coated groups compared to the levels in the uncoated group (Fig. 4E). Amongst the three coated groups, anti-CD31 Ab showed the most robust and reproducible improvements across all screening metrics (particularly CLDN and eNOS expression, which are closely related to barrier integrity and endothelial function), and therefore we prioritized anti-CD31 Ab for more detailed functional analyses, including µCT angiography, dextran leakage, and ex vivo and in vivo thrombogenicity. To confirm stability of the anti-CD31 coating over the duration of the bioreactor culture, we visualized the coated re-endothelialized scaffolds using fluorescence imaging against anti-CD31 (Supplementary Fig. 4). In addition, to assess whether these effects extended to human endothelial cells, we performed whole lung recellularization experiments using HUVECs. Consistent with the PCLS results, anti-CD31 Ab–preconditioned scaffolds showed more efficient re-endothelialization by HUVECs than uncoated scaffolds (Supplementary Fig. 5).

### Evaluation of vascular functionality in re-endothelialized vessels

**Fig. 5:**
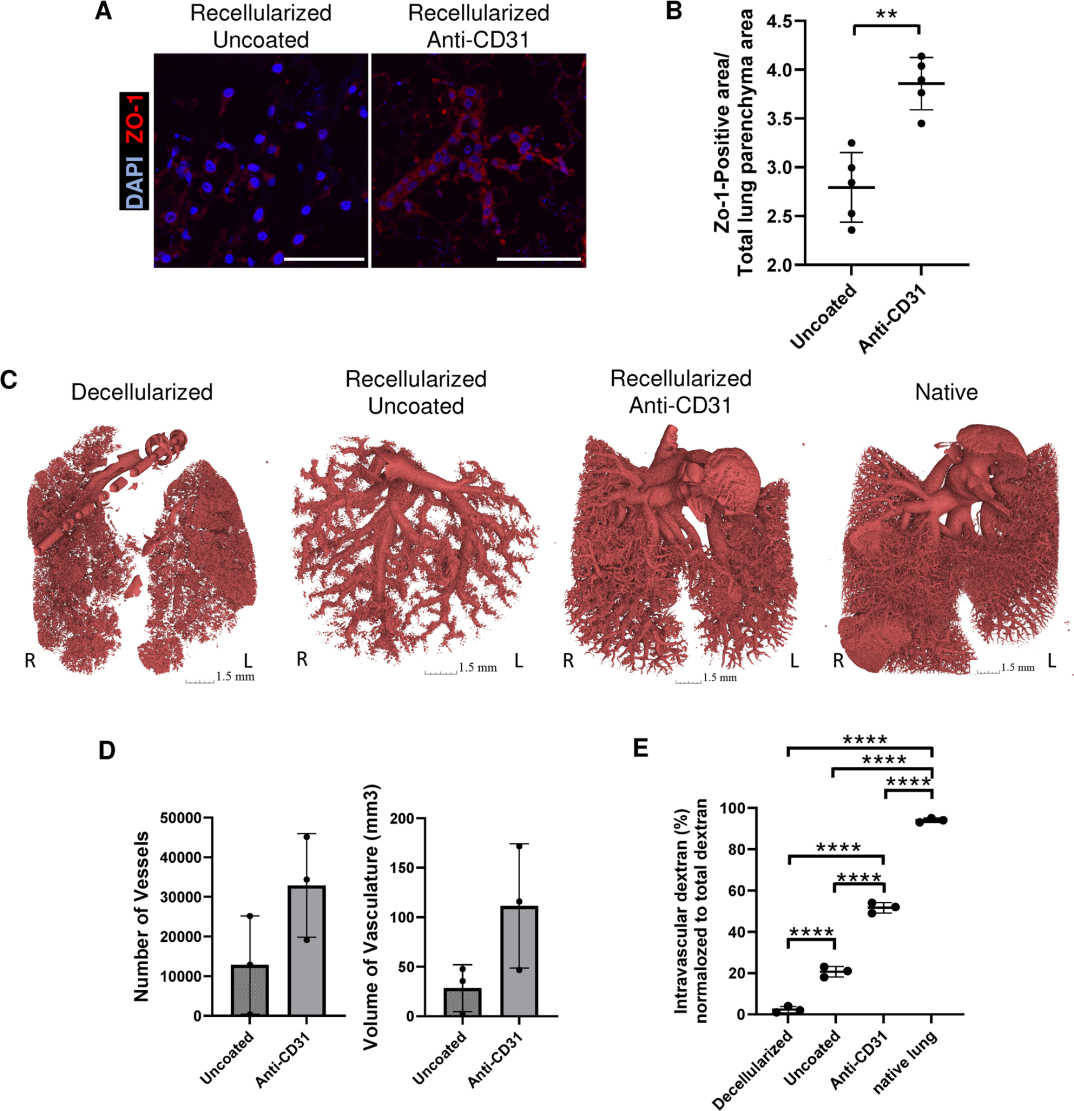
Assessment of pulmonary vascular regeneration following recellularization of anti-CD31 coated whole lung scaffolds. (**A**) Confocal images showing tight junction (ZO-1 (red)) immunostaining and nuclear staining with DAPI (blue). Scale bar = 100 μm. (**B**) Quantification of ZO-1 positive areas over total lung parenchyma. Each group *n* = 5, mean ± SD, **p < 0.01. (**C**) Micro-computerized tomography (µCT) image of decellularization scaffolds without re-endothelialization, uncoated scaffolds with subsequent recellularization, and CD31 Ab coated scaffolds with subsequent recellularization. (**D**) Quantification of number of vessels and volume of vasculature in the micro-CT image. Each group *n* = 3, mean ± SD. (**E**) Quantification of intravascular dextran volume drained from the pulmonary vein of each construct after perfusion of 500 kDa dextran via pulmonary artery. The results represent the mean of measurements taken from 3 technical replicates. Each number represents a biological replicate (independent whole lung scaffold). Each group *n* = 3, mean ± SD, ****p < 0.0001

Building on these findings, we proceeded to evaluate vascular endothelial function in grafts coated with the anti-CD31 antibody. Zonula occludens protein-1 (ZO-1) staining was performed to assess the state of tight junctions, revealing that the vascular endothelium was appropriately structured in the anti-CD31 Ab group (Fig. 5A and B). Angiography utilizing our previously established X-ray micro-computed tomographic (µCT) imaging [42] showed markedly enhanced vasculature in the anti-CD31 antibody precoated scaffold group (Fig. 5C). Representative images show clearly delineated branches of the vascular tree in the re-endothelialized anti-CD31 Ab preconditioned lungs and the native lungs with the contrast agent largely confined to the vascular lumen. In contrast the decellularized lung groups, there is observed leakage of the contrast agent into the parenchyma, resulting in very few visualized vessels. Although there were no significant statistical differences, the anti-CD31 Ab coated group tended to have more imaged blood vessels and volume than the uncoated group (Fig. 5D).

To estimate vascular endothelium leakage, a dextran perfusion assay was performed by injecting dextran through the pulmonary artery and measuring dextran discharged from the pulmonary vein. The ratio of dextran returned to the pulmonary veins to total dextran excretion (including excretion to the lung surface and trachea) was 2.3% ± 1.5 on average in decellularized lungs without re-endothelialization. Re-endothelialization (without any pre-treatment) increased the ratio to 20.6% ± 2.5. For the grafts pre-treated with anti-CD31, the ratio significantly increased to 51.6% ± 2.5 (*p* < 0.0001) demonstrating that the coating technique enhances vascular endothelial function in bioengineered lungs as measured by reduced dextran extravasation (Fig. 5E).

### Assessment of thrombogenicity in re-endothelialized constructs after blood perfusion

**Fig. 6:**
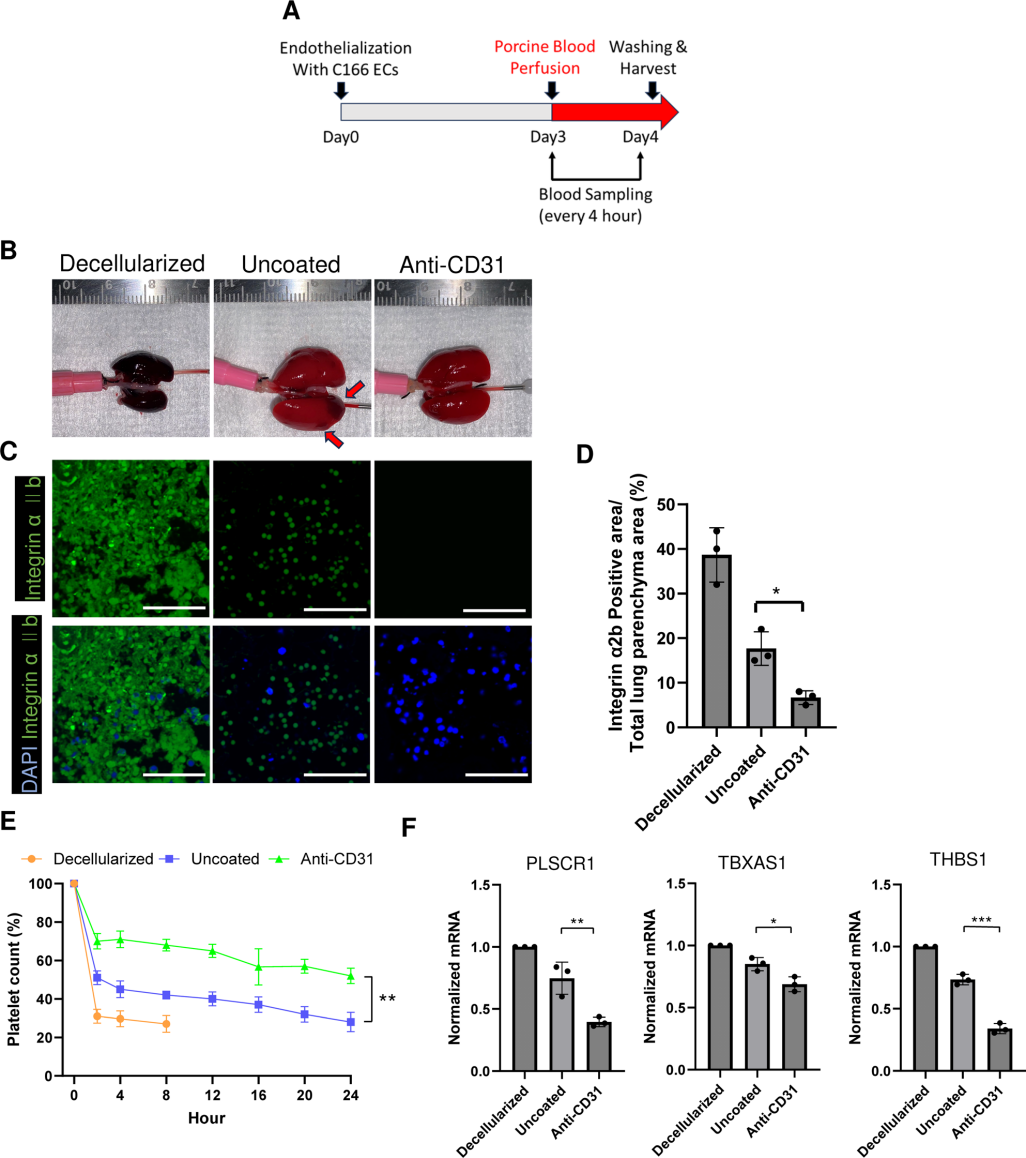
Evaluation of thrombogenicity in recellularized scaffolds following porcine blood perfusion. (**A**) Schematic showing experimental approach. (**B**) Macroscopic view of each scaffold after perfusion with porcine blood. Acellular scaffolds were used as a negative control. (**C**) Representative confocal images of harvested constructs stained with integrin αIIb (green). DAPI was used for detection of nuclei (blue). Scale bar = 50 μm. (**D**) Quantification of area of integrin αIIb positive areas. Each group *n* = 3, mean ± SD, **p < 0.01, versus Uncoated. (**E**) was harvested at the indicated time points, followed by platelet quantification. Each group *n* = 3 independent experiments. (**F**) Quantification of gene expression via RT-PCR analysis of *PLSCR1*,*TBXAS1* and*THBS1* (involved in platelet aggregation in blood-perfused constructs.*GAPDH* was used as a housekeeping gene. The results represent the mean of measurements taken from 3 technical replicates. Each number represents a biological replicate (independent whole lung scaffold). Each group *n* = 3, mean ± SD, *p < 0.05, **p < 0.01, ***p < 0.001, compared to the Uncoated group

Thrombus formation is a concern during the transplantation of bioengineered lungs. Therefore, we investigated the thrombogenicity of the anti-CD31 Ab-coated group compared to the uncoated group via perfusion of porcine blood into the bioengineered lungs (Fig. 6A). Although it would be ideal to use mouse blood, it was not feasible to acquire enough mouse blood for the perfusion studies. Blood was perfused into the bioengineered lung for 24 h in the perfusion bioreactor system from the PA. In the decellularized group without re-endothelialization, perfusion was discontinued after 8 h because the entire lung was occluded with thrombi. Thrombus formation was not observed in the anti-CD31 Ab group, whereas it was noted in the recellularized group without pre-treatment with anti-CD31 Ab (arrow, Fig. 6B). Immunostaining for integrin αIIb revealed considerable platelet aggregation in the decellularized and uncoated groups (Fig. 6C, D). In contrast, the anti-CD31 Ab group displayed a reduced number of activated platelets. Quantitative analysis confirmed that the αIIb⁺ area was 38.7 ± 6.1 in the decellularized group, 17.6 ± 3.8 in the uncoated group, and 6.7 ± 1.5 in the anti-CD31 Ab group, with significantly lower values in the anti-CD31 group compared with both the decellularized and uncoated groups (*p* = 0.0002 and *p* = 0.04, respectively; Fig. 6D). Furthermore, in the decellularized and uncoated lung groups, due to incomplete re-endothelialization of blood vessels, a marked decrease in platelet count was observed in the perfused blood at each time point compared to the initial time point (Fig. 6E). In contrast, decrease in platelet count was suppressed in the anti-CD31 Ab group during blood perfusion. At 24 h, platelet counts were reduced to 28.0 ± 5.0% i the uncoated group, whereas they were maintained at 52.0 ± 4% i the anti-CD31 Ab group (*p* = 0.003; Fig. 6E). Additionally, the expression analysis of thrombogenic genes (phospholipid scramblase 1 (*PLSCR1*), thromboxane A synthase 1 (*TBXAS1*), and thrombospondin 1 (*THBS1*)) demonstrated lower levels in the anti-CD31 Ab group compared to the other groups (Fig. 6F). These results demonstrated that anti-CD31 Ab-coated bioengineered lung suppresses thrombus formation.

### Post-transplant reperfusion of bioengineered mouse lung constructs

**Fig. 7:**
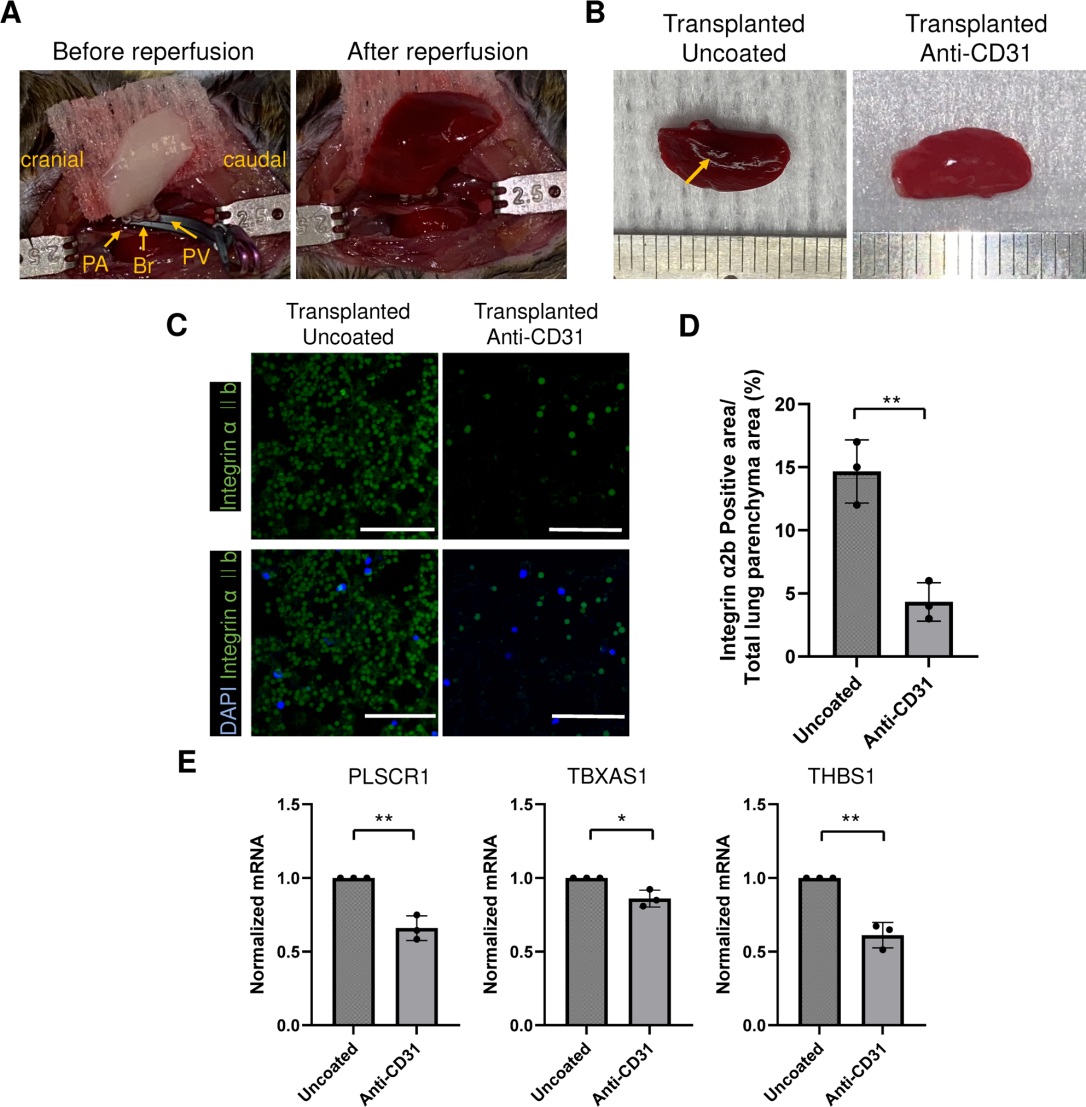
Evaluation of in vivo reperfusion of anti-CD31 Ab-coated re-endothelialized constructs. (**A**) Transplantation of re-endothelialized left lung constructs. PA; pulmonary artery, Br; bronchus, PV; pulmonary vein. (**B**) The representative gross images of harvested constructs after transplantation. (**C**) Representative confocal microcopy images of sections from harvested constructs stained with integrin αIIb (green) and nuclear DAPI (blue). Scale bar = 50 μm. (**D**) Quantification of integrin αIIb positive area. Each group *n* = 3, mean ± SD, **p < 0.01. (**E**) Quantification of gene expression via RT-PCR analysis of *PLSCR1*,*TBXAS1* and*THBS1* genes which are involved in platelet aggregation in blood-perfused constructs.*GAPDH* was used as a housekeeping gene. The results represent the mean of measurements taken from 3 technical replicates. Each number represents a biological replicate (independent whole lung scaffold). Each group *n* = 3, mean ± SD, *p < 0.05, **p < 0.01

Importantly, we investigated whether bioengineered lungs could be perfused in vivo in a short-term mouse transplant model. For proof of concept, we transplanted re-endothelialized left lung and evaluated function 30 min post-transplant. This was intentionally designed as a proof-of-concept study to verify blood vessel patency, confirm absence of immediate thrombosis, and demonstrate initial feasibility. The pulmonary artery, pulmonary vein, and main bronchus of the bioengineered mouse left lung were anastomosed to the left pulmonary artery, pulmonary vein, and main bronchus of the recipient mouse, respectively, and transplantation was performed under general anesthesia. After anastomosis of the bronchus and blood vessels was completed, the clamp on the anastomosed blood vessels was opened and reperfusion into the bioengineered lung was observed (Fig. 7A). After 30 min of perfusion, the mouse was euthanized and the left lung was removed. Thrombus formation continued to be observed in the uncoated group (arrow, Fig. 7B). In contrast, anti-CD31 Ab coated lungs showed broader areas of blood perfusion without thrombus formation. Immunostaining confirmed numerous integrin αIIb-positive immunostained cells in the uncoated group (Fig. 7C). In contrast, there were significantly fewer immunostained cells in the anti-CD31 Ab group. Quantification showed that the ratio of integrin αIIb positive area was 14.7% ± 2.5 in the.

uncoated group, while it was 4.3% ± 1.5 in the anti-CD31 Ab group (*p* = 0.004, Fig. 7D). This indicated that platelet aggregation was suppressed more effectively in the anti-CD31 Ab group than in the uncoated group. Gene expression analysis revealed a significant reduction in the expression of thrombogenic genes (*PLSCR1*, *TBXAS1*, and *THBS1*) in the anti-CD31 Ab group (*p* = 0.002, 0.01, and 0.001 respectively, Fig. 7E). Taken together, these results affirm that coating with anti-CD31 Ab enhances re-endothelialization of vascular endothelial cells and suppresses thrombogenicity of the bioengineered blood vessel.

## Discussion

In lung bioengineering, successful re-endothelialization requires targeted delivery of endothelial cells to line the decellularized vasculature. However, the intrinsic porosity of lung scaffolds limits the efficiency of the endothelialization process and problems often arise when these cells migrate outside the blood vessels or into the lung parenchyma during cell seeding and perfusion culture in a bioreactor [27] reducing intravascular retention and compromising uniform endothelial coverage. This is a major hindrance to ultimate graft functionality due to the high risk of acute thrombus formation resulting in early graft failure. Therefore, it is essential to minimize transmural escape of endothelial cells and maximize coordinated lateral migration along the luminal surface to form uniform endothelial coverage [39, 40, 49], thus preventing thrombus formation. Our objective herein was to pretreat acellular scaffolds with bioactive factors to both target the endothelial cells to the lining of pulmonary blood vessels and to improve the overall efficiency of endothelialization. We aimed to enhance the re-endothelialization of acellular mouse lung scaffolds via pretreatment of the scaffolds with coating agents that promote endothelial cell attachment and function. In vitro screening experiments using PCLS showed that among five selected candidates, scaffold pretreatment with fibronectin, REDV, and the anti-CD31 Ab resulted in improved adhesion, proliferation, and migration ability of vascular endothelial cells. Ex vivo whole lung recellularization experiments further showed a marked increase in cell attachment and scaffold cellular coverage in scaffolds pre-coated with CD31 Ab which also showed enhanced vascular endothelial function and suppression of thrombotic tendencies in blood perfusion and transplantation experiments.

Attachment and survival of endothelial cells depends on the cell-ECM interactions [50] and that ECM proteins including fibronectin, collagen, and binding partners such as integrins, mediate cell adhesion and survival [51, 52]. In addition to cell-ECM interactions, growth factors and cell-secreted proteins are required for cell adhesion, growth, and function [53]. One of the challenges in tissue bioengineering is adequate cell localization and function on biomaterials and biological scaffolds [16]. As such, surface modification, commonly achieved using adhesive molecules and growth factors, is often performed to improve cell activity on the surface of a material without changing the overall structural integrity of the tissue [54–56]. Surface modifiers have included antithrombotics, growth factors and ECM proteins such as heparin, VEGF, and fibronectin [27, 57–61]. These have been shown to promote endothelial cell function by improving adhesion, migration, survival and proliferative abilities, leading to prevention of platelet aggregation, in situ endothelialization and transmural capillary ingrowth. REDV has also been shown to contribute to microstructural changes of collagen fibers in acellular tissues leading to an antithrombotic effect [62].

Our results show that the binding of anti-CD31 Ab, (also known as anti-PECAM-1) endothelial cell antibody, to decellularized scaffolds improves the coverage of endothelial vessel wall, leading to the generation of a homogenous endothelial layer through the vascular network. This in turn results in the prevention of platelet activation and aggregation [63, 64]. Platelet endothelial cell adhesion molecule-1 (PECAM-1/CD31) is a transmembrane glycoprotein and belongs to the immunoglobulin superfamily (a subset of the cell adhesion molecule family (CAMs)) [65]. The utility of anti-CD31 Ab for scaffold functionalization has been evaluated in the context of the liver where it has been shown to enhance endothelialization of liver blood vessels. Ko et al. showed that pretreatment of decellularized livers with an anti-CD31 Ab, resulted in greater endothelialization of the scaffold resulting from increased initial anti-CD31 adherence to the vessel walls [64]. In another study, Kim et al. showed that anti CD31 Ab and anti CD31 aptamers potentiate cell survival and angiogenesis potential via integrin-Akt signaling cascades, eventually yielding good aligned vascular structures in bioengineered liver. Moreover, aptamer coated scaffolds enabled perfusion with blood for 2 h with reduced platelet adhesion ex vivo, and renewed liver function in a hepatic fibrosis rat model [26].

Several other scaffold-coating strategies have been investigated to specifically promote endothelialization of decellularized organs, including coatings with extracellular matrix (ECM) proteins such as collagen and fibronectin [17, 66, 67], as well as the use of integrin-binding peptides (e.g., RGD) and antibody-based adhesion ligands [68, 69]. Although ECM proteins provide a permissive surface for adhesion, their effects are nonspecific and do not actively support antithrombotic signaling. Similarly, peptide ligands such as RGD can enhance early endothelial attachment but have limited influence on endothelial activation state or platelet interactions under perfusion. In contrast, the anti-CD31 coating used in this study targets a pan-endothelial marker and may additionally engage CD31-dependent signaling pathways that promote junctional integrity, reduce inflammatory activation, and inhibit platelet activation [70–72].

In addition to scaffold coating, various other approaches have been developed to enhance endothelialization of decellularized scaffolds, including loading with platelet-rich plasma which provides sustained release of multiple growth factors and promotes M2 macrophage polarization, as demonstrated in pancreatic scaffolds [73]. Another strategy involves covalent functionalization with specific peptide sequences (EAbuK-YIGSR), which showed improved endothelial cell attachment and growth in heart valve applications through targeted cell-binding motifs [74]. Similarly, Spiller et al. demonstrated that macrophage-mediated modulation of engineered tissues enhances vascularization and healing, highlighting the importance of immune-cell interactions in scaffold design and endothelial function [75]. Additionally, chemical modification through genipin crosslinking has demonstrated enhanced vascularization and reduced inflammatory response in kidney scaffolds [76]. In contrast to these complex approaches, our anti-CD31 antibody coating method provides a straightforward, single-step solution that achieves robust endothelialization and reduced thrombogenicity without requiring chemical modifications or multiple treatment steps.

In the context of lung scaffolds, we speculate that the primary effects of the anti-CD31 coating are increased attachment and adherence of endothelial cells to the decellularized tissue, reduced apoptosis, and enhancement of endothelial cell function. The anti-CD31 antibody coating significantly increased the expression of key endothelial genes including *CLDN* (barrier integrity), *CD34* (intercellular interactions), and *eNOS* (angiogenesis), while also improving tight junction formation. Together, we believe that these molecular characteristics translated into the improved functional outcomes of barrier function and vessel integrity. Furthermore, the coating promoted better endothelial cell organization along blood vessels, increased proliferation (higher Ki-67 expression) and improved cell viability (reduced TUNEL-positive cells). This is in agreement with the literature, as CD31/Pecam-1 has been shown to have a key role in regulating apoptosis (via regulation of apoptosis-associated proteins including Akt, Bcl-2, Bcl-X, caspase 8, p38/JNK and MAPK [77]). It is important to distinguish that the proliferative and migratory effects observed with anti-CD31 coating represent physiological endothelial repair, not tumor-associated signaling. CD31 promotes endothelial stability and quiescence [70, 71], and does not activate pathways required for tumorigenesis, such as sustained proliferation or genomic instability [78]. Although the tumorigenic risk appears low, it is not impossible. Therefore, for translation, long-term studies will be needed to fully confirm safety [79].

We observe reduced thrombogenesis and platelet aggregation in anti-CD31/PECAM-1 coated scaffolds. At the molecular level, the anti-CD31 coating significantly reduced the expression of key thrombogenic genes including *PLSCR1*, *TBXAS1*, and *THBS1*, which are directly involved in platelet activation and aggregation pathways. Thus, the anti-CD31 coated scaffolds maintained better platelet counts in circulation and showed significantly reduced platelet aggregation (evidenced by decreased integrin αIIb staining). This is supported by the role of PECAM-1 in playing a key role in platelet adhesion and aggregation, inhibiting platelet aggregation and acting suppressively in thrombopoiesis [80]. In addition to the descriptive finding that anti-CD31 pretreatment reduced blood clot formation, the underlying mechanisms of this antithrombotic effect warrant further discussion. In this study, we did not directly assess the expression of major procoagulant or anticoagulant mediators such as tissue factor or thrombomodulin in the re-endothelialized cells, which represents a limitation. Nonetheless, prior studies have shown that CD31 engagement on endothelial cells can activate signaling pathways including activation of Immunoreceptor tyrosine-based inhibitory motif (ITIM)-mediated pathways, that promote endothelial survival, maintain barrier integrity, and support a quiescent, anti-inflammatory, and antithrombotic phenotype [70, 71, 81]. Alongside the improved endothelial coverage observed in our model, these effects may contribute to the maintenance of a more anticoagulant endothelial interface and reduced initiation of coagulation pathways. Moreover, CD31 is highly expressed on platelets and leukocytes, and its ligation has been reported to deliver inhibitory signals that attenuate platelet activation and leukocyte adhesion [72, 82]. Thus, the reduced clot formation observed in anti-CD31–treated lungs may result from a combination of enhanced endothelial integrity and CD31-mediated suppression of platelet and leukocyte activation at the blood–scaffold interface. Although these mechanistic considerations remain speculative, they provide a logical framework to explain the antithrombotic phenotype and point toward specific pathways for future investigation.

Our study is not without limitations. Experimental conditions we did not consider included combinations of factors which can be done to explore the synergistic effects of bioactive agents, physiological flow mimicking environments, longer term stability of the coating and potential remodelling of the ECM. Specific to the latter, while we do not expect any ECM remodeling or degradation during the time frame of our studies, longer-term evaluation of the re-endothelialized lungs needs to include potential ECM remodeling via assessment of structural proteins, (i.e. collagen), and enzymes, such as matrix metalloproteinase and lysyl oxidase.

We acknowledge that there is a possibility that the anti-CD31 antibody would result in an immune reaction. As such, for translation, immune dampening technologies such as PEGylation must be integrated into the methodology for future translation. PEGylation, which creates a protective hydrophilic shield that masks immunogenic sites, also increases the hydrodynamic radius of the antibodies, thereby increasing their stability [83, 84]. Additionally, with recent advances in aptamer technology, the use of anti-CD31 aptamers (single stranded oligonucleotides), which are by nature not considered immune reactive, may be equally valuable and cost effective, making them a more practical solution as a coating agent for clinical translation.

With respect to our ex vivo blood perfusion studies, the use of heparinized plasma-containing pig whole blood introduces potential issues. First, xenorecognition, as xenoreactive antibodies and complement can interact with mouse-derived endothelial cells [85–87]. However, because the perfusion period was short, the model primarily reflects platelet- and coagulation-driven thrombogenicity rather than complement-amplified immune responses. Nonetheless, this xenogeneic mismatch represents a limitation of the current system, and future studies using species-matched or immunologically modified blood will be important to clarify immune contributions [85, 86]. Second, the use of heparinized blood may partially mask thrombogenic differences due to heparin’s anticoagulant and platelet-modulating properties [88–90]. All groups were exposed to identical heparin levels, but future studies need to incorporate minimally anticoagulated or non-heparinized blood perfusion and measure coagulation parameters such as activated partial thromboplastin time (aPTT) and activated clotting time (ACT) [91, 92] to better isolate scaffold-dependent antithrombotic effects. And finally, although the native, non-decellularized lung represents the physiological gold standard for thromboresistance [93, 94], its inclusion as a direct control was beyond the scope of the present study. Native lungs would differ fundamentally in cellular composition and CD31-mediated inhibitory signaling [38, 70], and thus a direct comparison would not provide a mechanistically interpretable benchmark within this study design. Our aim was to examine the relative improvement conferred by anti-CD31 ab coating. As the field advances, systematic comparisons with intact lungs will be essential to establish how closely bioengineered constructs can approach physiological levels of thromboresistance.

We have predominantly used a mouse vascular endothelial cell line (C166). C166 is an endothelial cell line that was isolated from the yolk sac of a 12-day-old, mouse embryo and widely used and has endothelial cell characteristics [95]. Our preliminary findings with HUVECs suggest that the anti-CD31 coating results in a similar positive response in human endothelial cells. The lung vasculature however, is composed of several endothelial subpopulations [96, 97] which differ in integrin expression and adhesion signaling, likely influencing their interaction with ECM- coatings [98]. Although only C166 ECs and HUVECs were used in this study, CD31 is broadly expressed across pulmonary EC lineages [70, 72], suggesting that anti-CD31 Ab coating could engage multiple EC populations. Future studies examining subtype-specific adhesion and signaling will be important for fully understanding the applicability of our approach. Importantly, these studies will likely need to include a more clinically relevant cell source such as pluripotent derived ECs.

Finally, we used mouse scaffolds in this study because of their low cost and their stability due to their small individual differences, and we will continue to evaluate large animals in the future. Despite these limitations, we believe that pretreatment of acellular scaffolds with anti-CD31 antibody could be an avenue for future studies aimed at improving the quality of re-endothelialization.

## Conclusion

In this study, we showed that our re-endothelialization technique, combined with cell specific antibody conjugation, contributed to uniform coverage of endothelial cells within the vasculature of decellularized lung scaffold, leading to improved blood flow under continuous flow conditions. Importantly, pretreatment with anti-CD31 antibody resulted in the inhibition of blood clot formation. Thus, our results demonstrated that reinforcement of endothelial cell attachment by antibody conjugation contributes to improved vascular function and antithrombotic properties after transplantation. We present here a promising technique for creating better vascularized bioengineered lung constructs using pretreatment of decellularized scaffolds with anti-CD31 antibody.

## Supplementary Information

Below is the link to the electronic supplementary material.

Supplementary Material 1: Fig: 1 Decellularization of the mouse lungs. (A) Gross appearance of native lung and decellularized lung demonstrates the whitish coloration and preservation of the shape and size of the scaffold after the decellularization process. (B) Histological appearance after H&E staining of decellularized lung showing no cellular material compared to native lung. Scale bar = 100 μm. (C) Confocal microscopy images showing nuclear DAPI staining of native and decellularized lung tissues reveals total removal of nuclei from decellularized lung tissues. (D) DNA quantification of native lung and decellularized scaffolds indicating that only a negligible amount of DNA is retained compared to that in native lung. The results represent the mean of triplicate measurements. Each number (n=) represents a biological replicate (independent decellularized lung scaffold). Each group n = 6, mean ± SD, **p < 0.01. (E) Quantification of the attachment of Hoechst-labeled HUVECs on anti-CD31 coated PCLS coated with 12.5, 25, 50, or 100 µg/mL anti-CD31 Ab. Results represent the mean of measurements taken from 3 PCLS seeded with HUVEC cells from 3 independent experiments. (F) Quantification of HUVEC cell metabolic activity using cell growth assay using Cell Counting Kit-8 (CCK-8) revealed that precoating of PCLS increases ECs growth and metabolic activity (Optical Density (OD) measured at Day 1, 3 and 5) compared to ECs cultured on uncoated PCLS. The results represent the mean of measurements taken from 3 technical replicates. Each number (n=) represents a biological replicate (independent experiment). Each group n = 3, mean ± SD, *p < 0.05, **p < 0.01, ***p < 0.001, ****p < 0.0001.

Supplementary Material 2: Fig: 2 Comparison of Lung Composition and Mechanical Properties between Native and Decellularized Mouse Lung Scaffolds. (A) Representative histological images of Masson’s Trichrome, Verhoeff-van Gieson, and Alcian Blue staining demonstrate that decellularization preserves the extracellular matrix composition. Collagen (blue, first row), elastin (black fibers, second row), and sulfated glycosaminoglycans (sGAGs; light blue, third row) show no significant differences between native and decellularized mouse lungs. (B) Uniaxial tensile testing indicates that decellularization does not significantly alter the stress–strain characteristics of the lung scaffold. Solid lines represent the fitted curves, and dashed lines denote the 95% confidence intervals for each group.

Supplementary Material 3: Fig3: Quantification of the attachment of Hoechst-labeled c166 mouse endothelial cells on coated PCLS for optimization of concentration of each coating candidate. For each factor, results represent the mean of measurements taken from 3 PCLS seeded with c166 cells from 3 independent experiments. Each group n = 3, mean ± SD, *p < 0.05, **p < 0.01, ***p < 0.001, ****p < 0.0001.

Supplementary Material 4: Fig. 4. Retention of anti-CD31 coating. (A) Representative confocal images of decellularized PCLS disks coated with 12.5, 25, 50, or 100 µg/mL anti-CD31 Ab visualized in green. (B) Representative confocal images showing retention of anti-CD31 antibody coating (green) post whole mouse lung C166 re-endothelialization on uncoated (left) and coated (right) whole lung scaffolds. Images are representative of n = 3 biological replicates. Scale bar = 100 μm.

Supplementary Material 5: Endothelialization of whole lung scaffolds with HUVECs. Hematoxylin and eosin (H&E) stained images of whole mouse lungs re-endothelialized with HUVECs with (top row) and without (bottom row) anti-CD31 coating.

Supplementary Material 6: Fig. 6. Reduced apoptosis in anti-CD31 coated re-endothelialized lungs. (A) Representative confocal images showing retention of Caspase 3 staining (red) and DAPI nuclear staining (blue) post whole mouse lung C166 reendothelialization on uncoated (left) and coated (right) whole lung scaffolds. Images are representative of n = 3 biological replicates. Quantification of Caspase 3 staining. Results represent the mean of measurements taken from 3 images per lung seeded with c166 cells from 3 independent experiments. (Each number (n=) represents a biological replicate (re-endothelialized whole lung scaffold). Each group n = 3, mean ± SD, *p < 0.05.

## Data Availability

The data that support the findings of this study are available from the author upon reasonable request.

## Abbreviations

Ab: Antibody
LTx: Lung transplantation
ECM: Extracellular matrix
ECs: Endothelial cells
SDS: Sodium dodecyl sulfate
PA: Pulmonary artery
PV: Pulmonary vein
dH2O: Distilled water
RT: Room temperature
PCLS: Precision cut lung slices
EDC: 1-(3-Dimethylaminopropyl)-3-ethylcarbodiimide hydrochloride
NHS: N-Hydroxysuccinimide
DMEM: Dulbecco’s Modified Eagle’s Medium
EGM: Endothelial cell growth medium
HUVEC: Human umbilical vein endothelial cells
CCK-8: Cell Counting Kit-8
µCT: Micro-computed tomographic
PBS: Phosphate buffered saline
OD: Optical Density
CLDN: Claudin
eNOS: Endothelial nitric oxide synthase
DAPI: 4’,6-diamidino-2-phenylindole
TUNEL: Terminal deoxynucleotidyl transferase (TdT)-mediated dUTP nick end labelling
ZO-1: Zonula occludens protein-1
PLSCR1: Phospholipid scramblase 1
TBXAS1: Thromboxane A synthase 1
THBS1: Thrombospondin 1
qRT-PCR: Quantitative reverse transcription-polymerase chain reaction
PECAM: Platelet endothelial cell adhesion molecule
CAMs: Cell adhesion molecule
