# Supplementary figures and images for: Anti-CD31 antibody preconditioning for enhancement of endothelial cell capture and vascularization: a novel strategy for bioengineering lung scaffolds
Source: J Biol Eng. 2026 Jan 8;20:27. doi: 10.1186/s13036-025-00593-x (PMC12874679; doi:10.1186/s13036-025-00593-x)

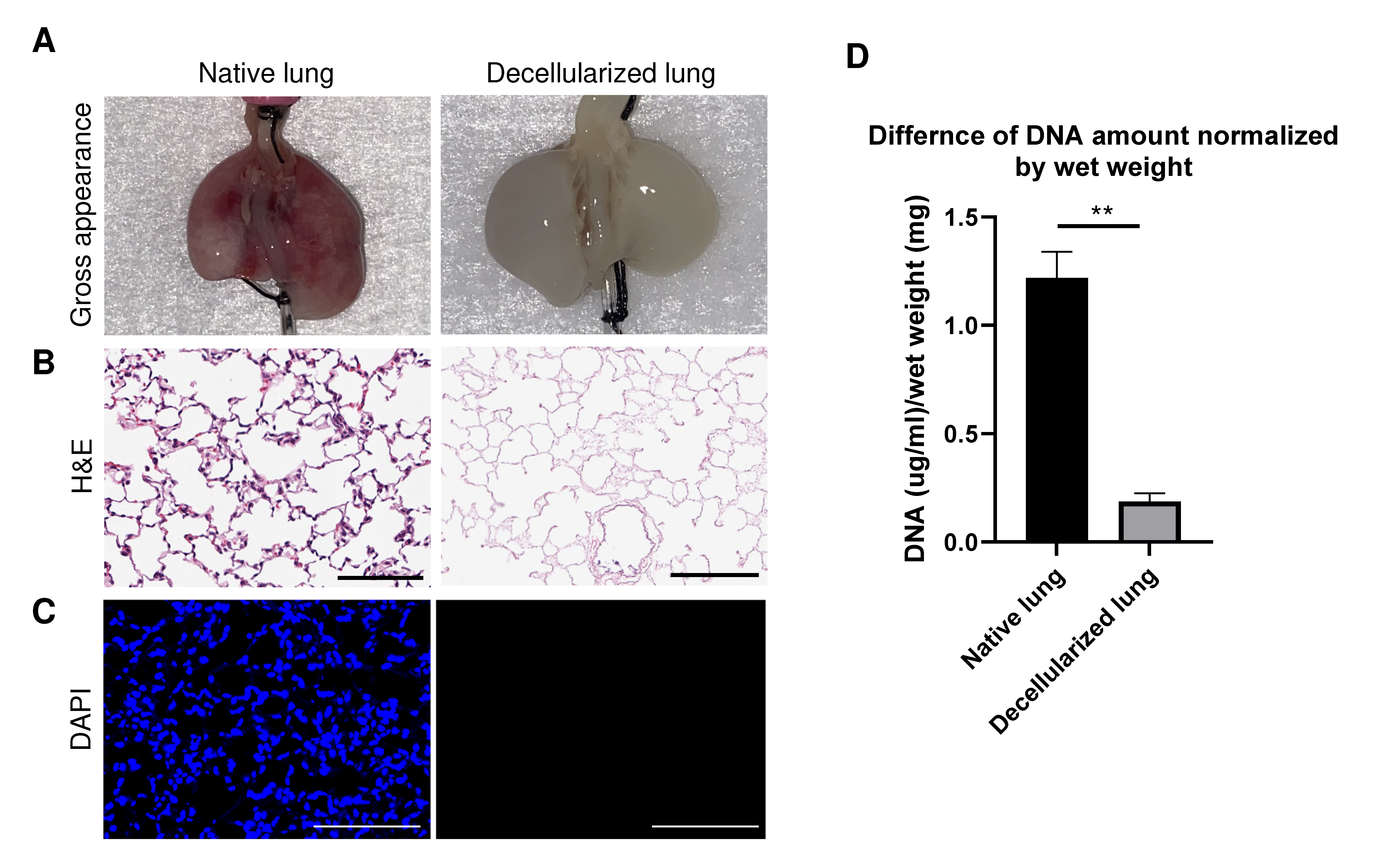

Supplement: Supplementary file 1 — Supplementary Material 1: Fig: 1 Decellularization of the mouse lungs. (A) Gross appearance of native lung and decellularized lung demonstrates the whitish coloration and preservation of the shape and size of the scaffold after the decellularization process. (B) Histological appearance after H&E staining of decellularized lung showing no cellular material compared to native lung. Scale bar = 100 μm. (C) Confocal microscopy images showing nuclear DAPI staining of native and decellularized lung tissues reveals total removal of nuclei from decellularized lung tissues. (D) DNA quantification of native lung and decellularized scaffolds indicating that only a negligible amount of DNA is retained compared to that in native lung. The results represent the mean of triplicate measurements. Each number (n=) represents a biological replicate (independent decellularized lung scaffold). Each group n = 6, mean ± SD, **p < 0.01. (E) Quantification of the attachment of Hoechst-labeled HUVECs on anti-CD31 coated PCLS coated with 12.5, 25, 50, or 100 µg/mL anti-CD31 Ab. Results represent the mean of measurements taken from 3 PCLS seeded with HUVEC cells from 3 independent experiments. (F) Quantification of HUVEC cell metabolic activity using cell growth assay using Cell Counting Kit-8 (CCK-8) revealed that precoating of PCLS increases ECs growth and metabolic activity (Optical Density (OD) measured at Day 1, 3 and 5) compared to ECs cultured on uncoated PCLS. The results represent the mean of measurements taken from 3 technical replicates. Each number (n=) represents a biological replicate (independent experiment). Each group n = 3, mean ± SD, *p < 0.05, **p < 0.01, ***p < 0.001, ****p < 0.0001. [file 13036_2025_593_MOESM1_ESM.tif]

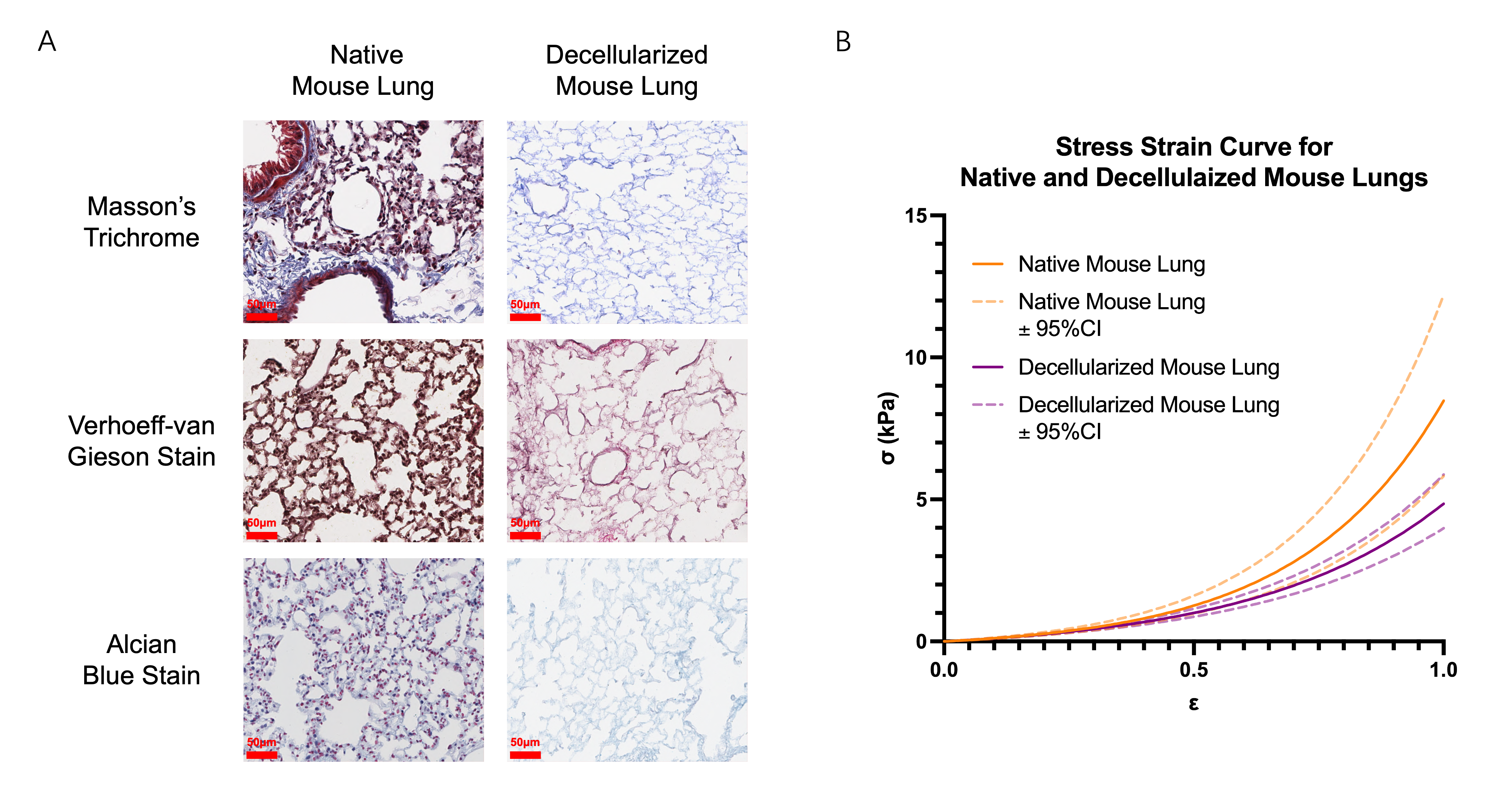

Supplement: Supplementary file 2 — Supplementary Material 2: Fig: 2 Comparison of Lung Composition and Mechanical Properties between Native and Decellularized Mouse Lung Scaffolds. (A) Representative histological images of Masson’s Trichrome, Verhoeff-van Gieson, and Alcian Blue staining demonstrate that decellularization preserves the extracellular matrix composition. Collagen (blue, first row), elastin (black fibers, second row), and sulfated glycosaminoglycans (sGAGs; light blue, third row) show no significant differences between native and decellularized mouse lungs. (B) Uniaxial tensile testing indicates that decellularization does not significantly alter the stress–strain characteristics of the lung scaffold. Solid lines represent the fitted curves, and dashed lines denote the 95% confidence intervals for each group. [file 13036_2025_593_MOESM2_ESM.tif]

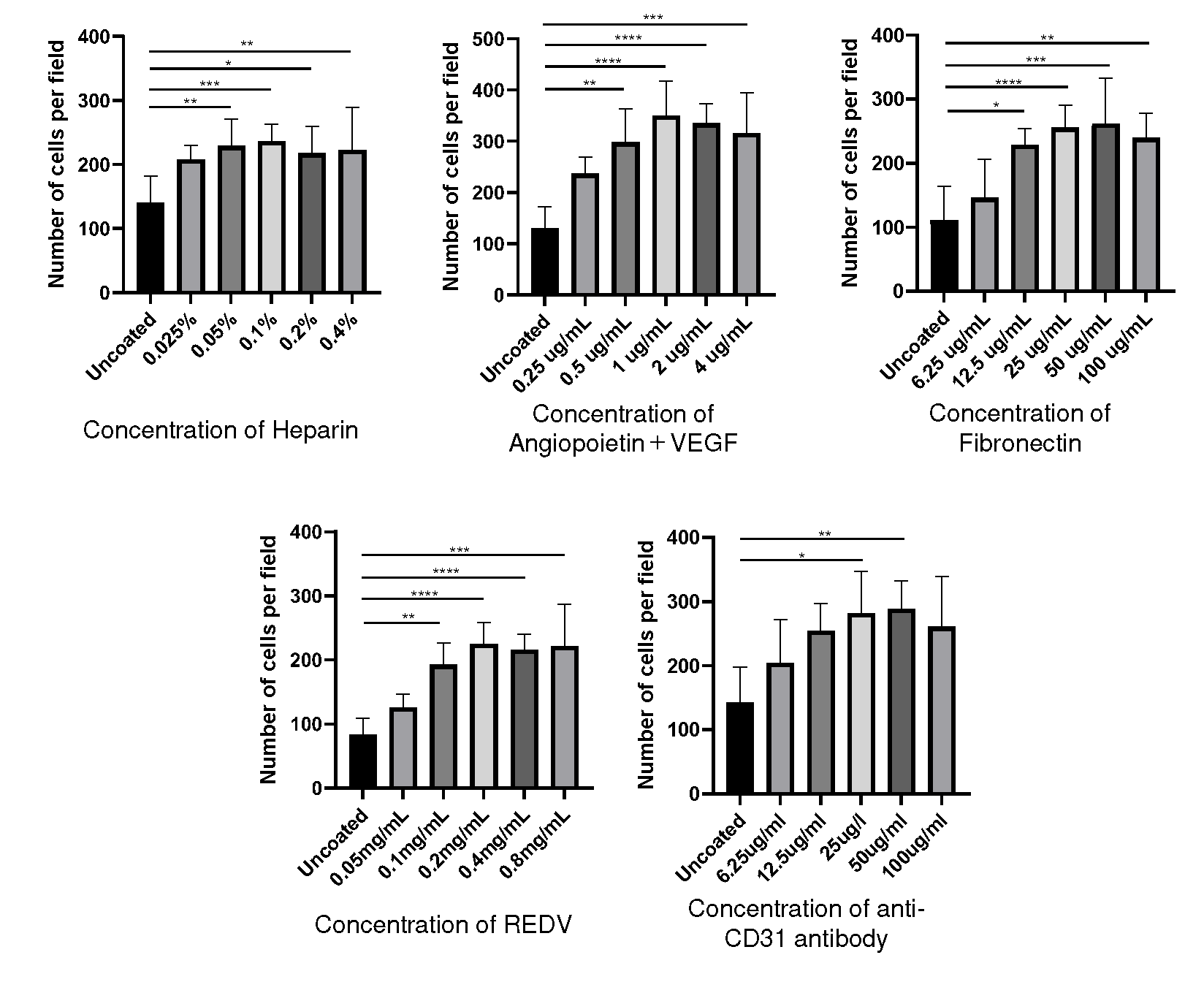

Supplement: Supplementary file 3 — Supplementary Material 3: Fig3: Quantification of the attachment of Hoechst-labeled c166 mouse endothelial cells on coated PCLS for optimization of concentration of each coating candidate. For each factor, results represent the mean of measurements taken from 3 PCLS seeded with c166 cells from 3 independent experiments. Each group n = 3, mean ± SD, *p < 0.05, **p < 0.01, ***p < 0.001, ****p < 0.0001. [file 13036_2025_593_MOESM3_ESM.tif]

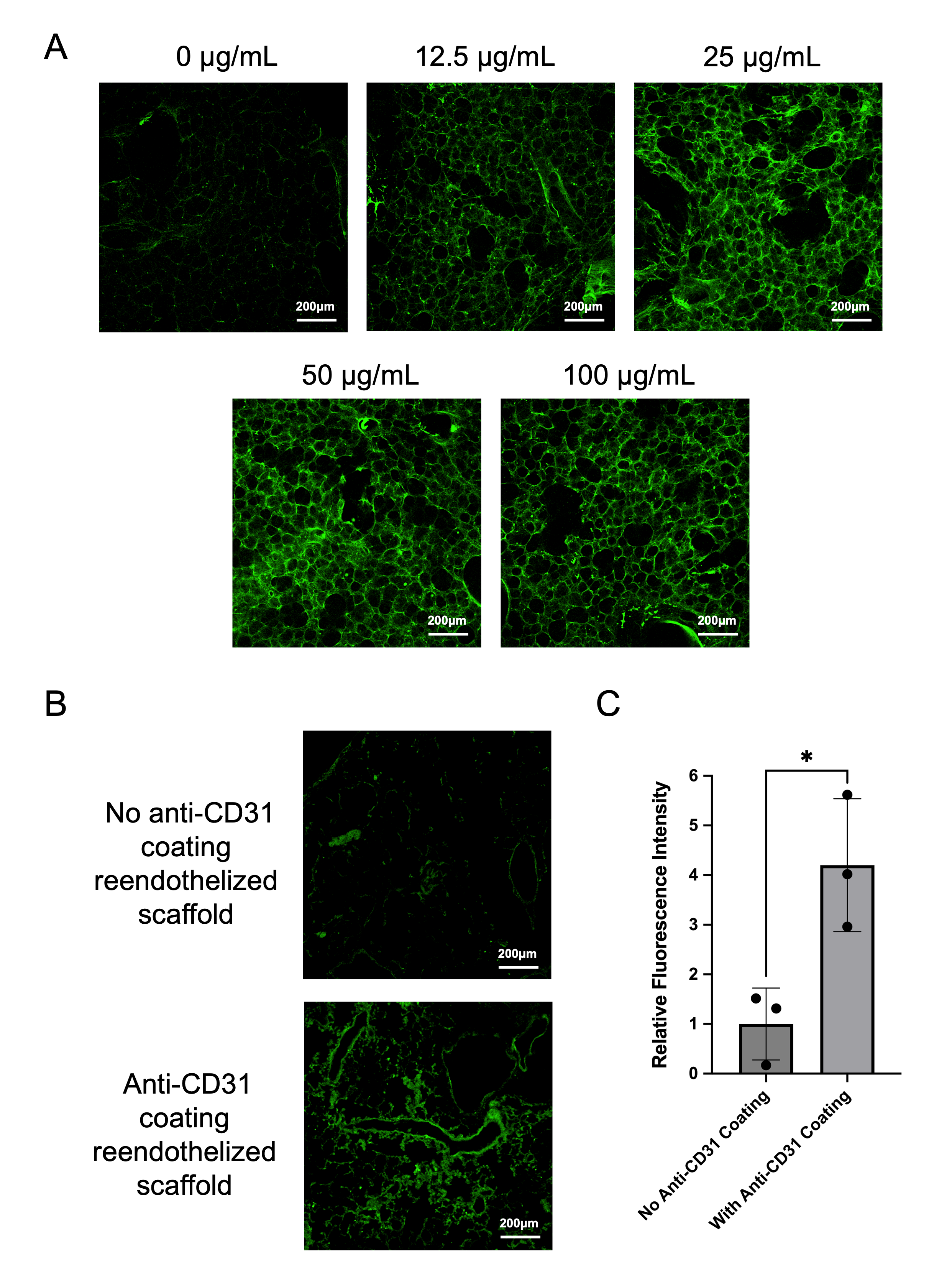

Supplement: Supplementary file 4 — Supplementary Material 4: Fig. 4. Retention of anti-CD31 coating. (A) Representative confocal images of decellularized PCLS disks coated with 12.5, 25, 50, or 100 µg/mL anti-CD31 Ab visualized in green. (B) Representative confocal images showing retention of anti-CD31 antibody coating (green) post whole mouse lung C166 re-endothelialization on uncoated (left) and coated (right) whole lung scaffolds. Images are representative of n = 3 biological replicates. Scale bar = 100 μm. [file 13036_2025_593_MOESM4_ESM.tif]

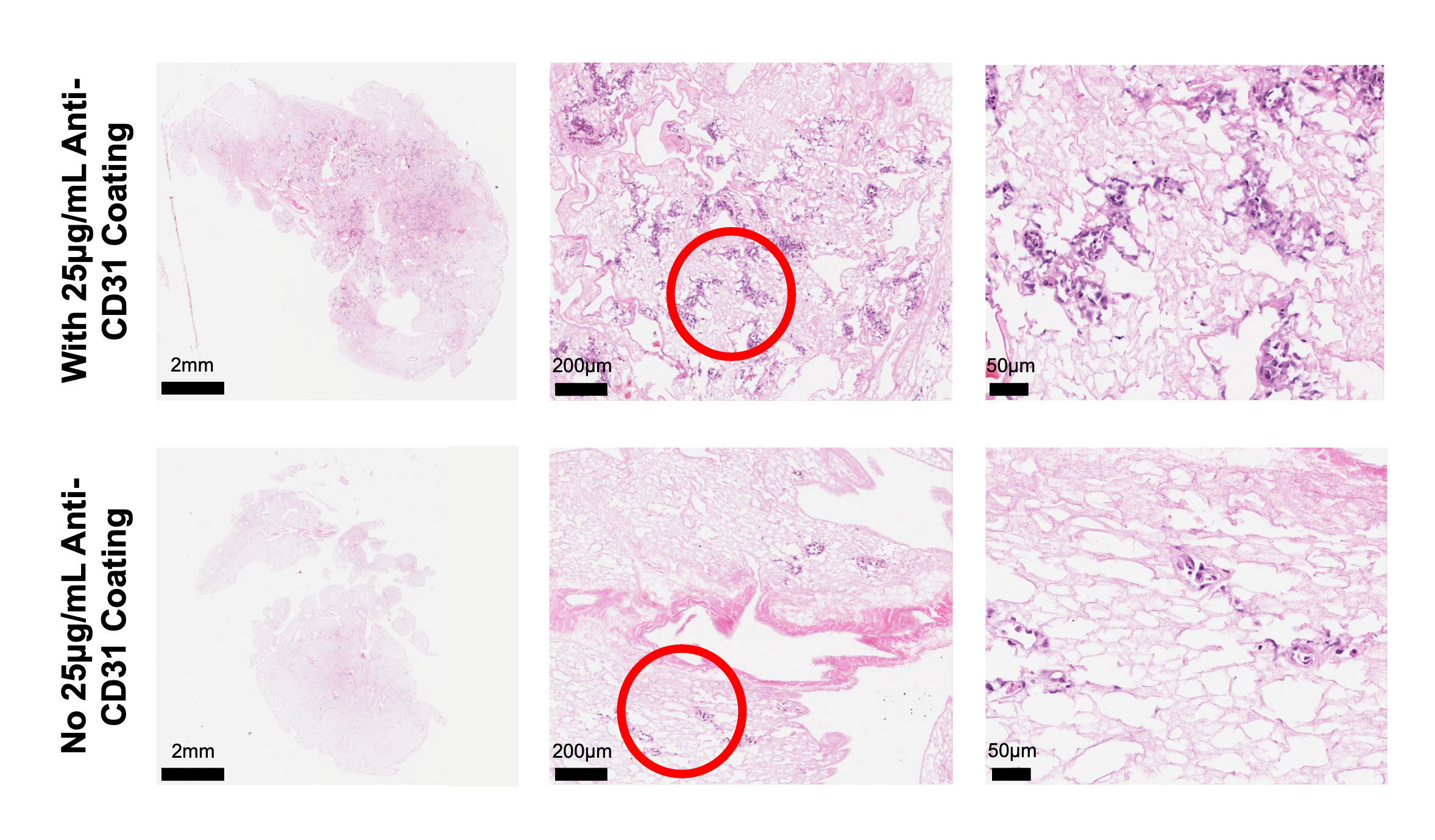

Supplement: Supplementary file 5 — Supplementary Material 5: Endothelialization of whole lung scaffolds with HUVECs. Hematoxylin and eosin (H&E) stained images of whole mouse lungs re-endothelialized with HUVECs with (top row) and without (bottom row) anti-CD31 coating. [file 13036_2025_593_MOESM5_ESM.tif]

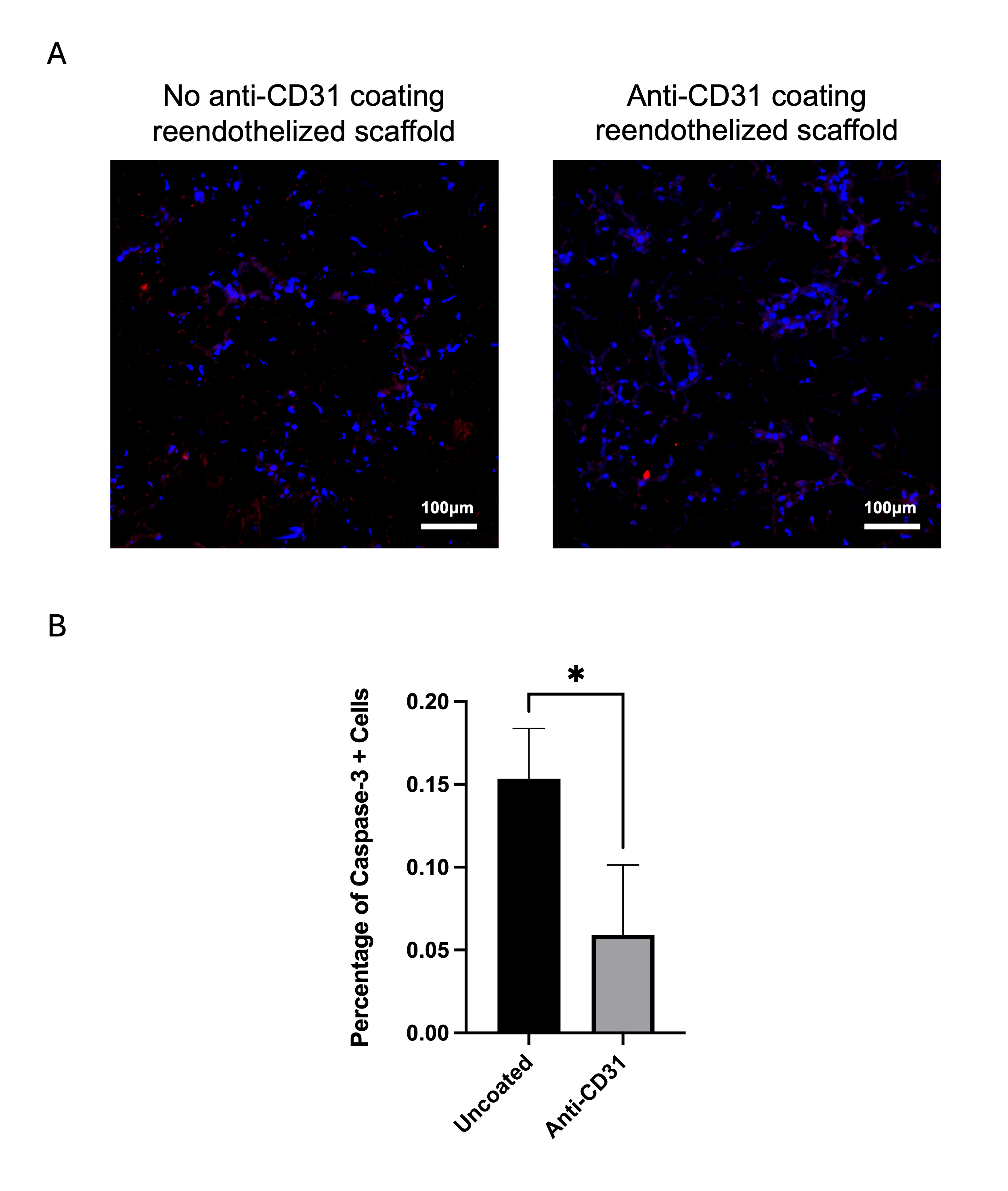

Supplement: Supplementary file 6 — Supplementary Material 6: Fig. 6. Reduced apoptosis in anti-CD31 coated re-endothelialized lungs. (A) Representative confocal images showing retention of Caspase 3 staining (red) and DAPI nuclear staining (blue) post whole mouse lung C166 reendothelialization on uncoated (left) and coated (right) whole lung scaffolds. Images are representative of n = 3 biological replicates. Quantification of Caspase 3 staining. Results represent the mean of measurements taken from 3 images per lung seeded with c166 cells from 3 independent experiments. (Each number (n=) represents a biological replicate (re-endothelialized whole lung scaffold). Each group n = 3, mean ± SD, *p < 0.05. [file 13036_2025_593_MOESM6_ESM.tif]
